# Attitudes and perceptions of mothers towards childhood vaccination in Greece: lessons to improve the childhood COVID-19 vaccination acceptance

**DOI:** 10.3389/fped.2022.951039

**Published:** 2022-08-25

**Authors:** Georgia Fakonti, Andria Hadjikou, Eleana Tzira, Maria Kyprianidou, Konstantinos Giannakou

**Affiliations:** Department of Health Sciences, School of Sciences, European University Cyprus, Nicosia, Cyprus

**Keywords:** vaccination, immunization, attitudes, mothers, children, Greece, COVID-19

## Abstract

**Background:**

Maternal attitudes and beliefs have been shown to influence childhood vaccination coverage, resulting in under-vaccination, non-vaccination, and vaccination delay. This study aimed to investigate the mothers' attitudes and perceptions about vaccination for their children in Greece.

**Methods:**

This was an online cross-sectional study, conducted from 4 April to 8 June 2020. A self-administered questionnaire was used to collect information about mothers' and their children's socio-demographic characteristics, previous vaccination behavior, and mothers' attitudes and perceptions about childhood vaccination. Participants included adult mothers with at least one minor child.

**Results:**

One thousand eight hundred eighty-five mothers participated, with the majority (91.7%) believing in the usefulness of vaccines and that vaccines protect children from serious and life-threatening diseases. A larger percentage of mothers with higher educational attainment agreed/absolutely agreed that all vaccinations provided by the National Vaccination Program must be offered to their children (91.6%) (*p* = 0.02) and that vaccines protect children from serious and life-threatening diseases (92.9%) (*p* = 0.01). Significant more married/in cohabitation and not single-parent mothers agreed that vaccines are safe (53.5% and 53.4%, respectively). There were also several significant associations between maternal attitudes toward childhood vaccination and previous maternal vaccination practices [(e.g., adherence to recommended vaccination dosages (all *p*-values < 0.01), vaccination delays (all *p*-values < 0.05), and vaccination during pregnancy (all *p*-values < 0.01)].

**Conclusion:**

Maternal attitudes and perceptions toward childhood vaccination are significantly influenced by sociodemographic factors and maternal vaccination practices. Revealing those is essential for public health officials in developing future strategies to improve childhood vaccination coverage and acceptance of new vaccines such as the COVID-19 vaccine.

## Introduction

Vaccination is undeniably a public health success story for disease eradication (1). Vaccines have been administrated since 1796, preventing millions of deaths and adverse health outcomes (2). They significantly reduced healthcare costs due to the decrease in hospitalizations and disease prevalence (3). Thus, vaccines are considered a cost-effective and life-saving tool (4, 5). More than 200 years after the first vaccine development, the ongoing public health crisis from COVID-19 also highlights the importance of investing in vaccine development, since COVID-19 vaccination contributed to the reduction of hospitalizations, death rate, and adverse health outcomes (6, 7).

The failure of current treatments for bacterial infections, due to antibiotic resistance (8–11), and the burden of infectious diseases renders vaccine development a priority to avert severe infections (12–15). The development of new vaccines is a long complex process, with several attempts to create vaccines for fatal diseases such as AIDS, malaria, and a range of parasitic infections being unsuccessful (16–18). Although vaccine development is a significant milestone for humanity, the public may delay or refuse vaccination with available vaccines: vaccine hesitancy. Vaccine hesitancy was raised during the last decades and is influenced by several determinants including ethnicity, religious, socio-economic factors, beliefs, knowledge, and safety and efficacy concerns (19–24). In addition, unfavorable vaccine incidences could reduce vaccine trust (25). Consequently, specific vaccination rates may not attain adequate coverage to prevent epidemics (26–30).

Childhood vaccination reduces the mortality rate, although some children are unvaccinated or under-vaccinated (31–33). Of note, during the COVID-19 pandemic some childhood vaccinations were delayed or missed (34–36). Specifically, after March 2020 considerably fewer children received the vaccines against diphtheria, pertussis, tetanus, and measles compared to 2019 (37, 38). Several reasons contributed to this decline including the lack of access to health services due to lockdown restrictions, and the shortage of health care staff during the COVID-19 pandemic (39). Also, there is an increasing concern that the pandemic regulations influenced health care professionals' communications with parents, hence limiting routine vaccination promotion (40).

Except for the COVID-19 pandemic, parental hesitancy toward childhood vaccination affects childhood vaccination uptake (26, 41–49). Attitudes and perceptions of parents toward vaccines may influence parental decisions to vaccinate their child/children. Parental vaccination behavior and experiences may result in childhood vaccination delays or refusal (50, 51). In addition, negative opinions toward vaccination may enhance the risk of unvaccinated children and increase the likelihood of disease outbreaks. Recently, outbreaks of vaccine-preventable disease were observed in Europe due to inadequate vaccination rates (27); hence it is essential to estimate the parental attitudes toward childhood vaccinations. Mothers seem to have a prime role in childcare (52), therefore, the present study aimed to evaluate the attitudes and perceptions of mothers in Greece concerning the vaccination of their child/children. Understanding maternal attitudes and beliefs regarding the vaccination of their children is crucial, especially during the COVID-19 pandemic.

## Methods

### Study design

This was an online cross-sectional study. This study reported following the Strengthening the Reporting of Observational Studies in Epidemiology (53).

### Study duration and study setting

This study was conducted from 4 April 2020 to 8 June 2020. The population of interest was mothers 18 years old and older with at least one minor child (<18 years old), who were living in the four geographical areas of Greece (Attica, Central Greece, North Greece, and Crete/Aegean Islands).

### Sample size

The sample size needed for this study was calculated using the formula, $n=\frac{z^{2}\times P\times \left(1-p\right)}{d^{2}}$. Assuming a vaccination coverage of 90%, based on a literature review on the topic, a z equal to 1.96 under a 95% confidence interval and a precision of 2%, a sample size of ~864 individuals was required to estimate this (54).

### Sampling method

Due to the quarantine restrictions resulting from the ongoing COVID-19 pandemic, a convenience sampling method was used, with a potential effect on sampling possibilities. The questionnaire was administered using Google Forms and dispersed using social media applications (e.g., Facebook, Instagram), instant messaging apps (e.g., WhatsApp, Viper), and social networking sites (e.g., LinkedIn), to gather a sample from all geographical areas of Greece [Attica (46.8%) of the total Greek population, Central Greece (13.0%), North Greece (29.0%) and Crete/Aegean Islands (11.2%)]. Despite the non-probabilistic sampling approach used, we have managed to recruit women of different ages among the Greek mainland and islands, ensuring a relatively representative sample of the adult female Greek population.

### Survey instrument

The self-administered questionnaire in the Greek language was specially designed after an extensive review of relevant literature and included questions regarding mothers' socio-demographic characteristics, children's characteristics, general information about vaccination practices, and attitudes and perceptions of mothers regarding childhood vaccination (Supplementary File). The survey was pilot tested by 50 mothers for face validity and comprehensibility before study commencement. The pilot study sample was not included in the sample of the study. We have also calculated the Cronbach's alpha coefficient, which it was 0.92 indicating a strong internal consistency.

### Socio-demographic characteristics of mothers and their children

The first part of the questionnaire included questions regarding maternal socio-demographic characteristics (i.e., age, employment status, educational level, and marital status) and children's characteristics (i.e., age, and gender). Age was reported in years. Geographical area was recorded as Attica, Central Greece, North Greece, and Crete/Aegean Islands while area of residence was classified as urban and rural. Marital status was recorded as never married, married/ in cohabitation, or separated/divorced/widowed. Single parent family was evaluated using a binary question (Yes vs. No). Educational level was classified into three categories: primary education (participants who completed only primary school: < 7 years of schooling), secondary education (participants who completed middle or high school: 7–12 years of schooling), and higher education (participants who have a university degree: >12 years of schooling). Employment status was recorded as unemployed, private, and state or freelance employee. Income status was described using the monthly income and was classified as (i) no income; (ii) low (<1,101 per month); (iii) middle (1,101–1,500 per month); and (iv) high (>1,500 per month). Moreover, characteristics about the children included the number of children, the age, and the gender.

### Vaccination attitudes and perceptions

This part of the questionnaire assessed maternal attitudes and perceptions toward childhood vaccination, using 14 Likert-scale questions (Q1-Q14) on a five-point rating scale (1 = absolutely disagree, 2 = disagree, 3 = neither disagree nor agree, 4 = agree, and 5 = absolutely agree).

### Previous vaccination behavior

The questionnaire also included information about previous vaccination practices, including adherence to the prescribed doses as indicated by the local recommendations (Yes vs. No) and delay of vaccination (Yes vs. No). The question used to report the reasons of possible delay of vaccination was “If you have delayed your child/children vaccination, what were the main reasons?” with possible answers being illness, lack of clear information, paediatrician's suggestion, fear of side effects of the vaccine, increased cost of vaccines, increased cost of medical visit, long distance from the vaccination site or other. Vaccination during pregnancy was reported using the question “Have you been vaccinated during your pregnancy?” (Yes vs. No). Participants' general knowledge toward vaccination was assessed using a 12-item scale, but these analyses are reported elsewhere (55).

### Statistical analysis

Descriptive statistics included frequencies (n)/percentages (%) for categorical variables and mean/ standard deviation (SD) or median values/interquartile range (IQR) for continuous variables with normal or skewed distribution, respectively. The skewness of distribution was used to check the normality of numeric variables using the Shapiro–Wilk normality test. Chi-squared test, one-way analysis of variance (ANOVA), and non-parametric tests including Kruskal–Wallis were used to test significant differences between variables. Bonferroni correction test was used to address the problem of multiple comparisons. Bar charts were constructed to present the maternal attitudes and perceptions toward their children's vaccination. All statistical tests performed were two-sided, with the statistical significance level set at α = 0.05. Statistical analysis was conducted using STATA 14.0 (Stata Corp, College Station, TX, USA).

### Ethics

This study was conducted according to the Declaration of Helsinki guidelines and all procedures involving research study participants were approved by the Cyprus National Bioethics Committee (CNBC) (EEBK EΠ 2020.01.82). The survey was voluntary, and no incentive was offered. All the participants were informed about the study's aim and objectives before participating on the first page of the questionnaire. The respondents needed to confirm their willingness to participate voluntarily by answering a “Yes/No” question on an electronic mandatory informed consent form before being allowed to complete the online self-reporting questionnaire.

## Results

### Participants' characteristics

A total of 1,885 mothers, who live in the four geographical areas of Greece, completed the online questionnaire (Supplementary Figure 1). The mean age of the participants was 36.3 years old (SD = 5.0 years old) (Table 1). Almost half of the participants (46.8%) were residents of the Attica region (including Athens and its suburbs) and 87.0% were living in an urban area. The overwhelming majority were married (95.1%), while 5.1% were single parents. In addition, 73.5% of them had completed a higher education, 39.4% were private employees, and 56.6% had a high monthly salary. Participants reported 2,950 children with a median age of 42 months (IQR: 24–72 months), among whom 51.8% were boys. The majority of the mothers strictly adhered to the prescribed dosage as indicated by the local recommendations for each vaccine (94.3%), have delayed their child/children vaccination (51.5%) and have not been vaccinated during pregnancy (75.7%) (Table 1).

**Table 1:** Characteristics of the mothers and their children as well as previous vaccination behavior characteristics.

Total number of mothers [N (%)]		1,885 (100)
Mean age of mothers [years (SD)]^**a**^		36.3 (5.0)
Gender of children [N (%)]^**b**^	Boys	1,529 (51.8)
	Girls	1,421 (48.2)
Median age of children [months (IQR)]^**b**^		42 (24–72)
Geographical region of residence [N (%)]^**c**^	Attica	883 (46.8)
	Central Greece	245 (13.0)
	North Greece	546 (29.0)
	Grete/Aegean Islands	211 (11.2)
Area of residence [N (%)]^**d**^	Urban	1,577 (87.0)
	Rural	235 (13.0)
Marital status of mother [N (%)]^**e**^	Unmarried	28 (1.5)
	Married/In cohabitation	1,789 (95.1)
	Divorced/Separated/Widowed	64 (3.4)
Single parent family [N (%)]^**a**^	No	1,785 (94.9)
	Yes	97 (5.1)
Educational attainment of mother [N (%)]^**a**^	Primary education	10 (0.6)
	Secondary education	488 (25.9)
	Higher education	1,384 (73.5)
Employment status of mother [N (%)]^**a**^	Unemployed	463 (24.6)
	State employee	349 (18.5)
	Private employee	740 (39.4)
	Freelance	330 (17.5)
Income status of mother [N (%)]^**f**^	No income	48 (2.6)
	Low (< €1,1001/month)	276 (14.9)
	Medium (€1,101–1,500/month)	481 (25.9)
	High (> €1,500/month)	1,049 (56.6)
Do you strictly adhere to the prescribed dosage as indicated by the local recommendations for each vaccine? [N (%)]^**g**^	No Yes	107 (5.7) 1,770 (94.3)
Have you ever delayed your child/children vaccination? [N (%)]^**h**^	No Yes	910 (48.5) 966 (51.5)
If you have delayed your child/children vaccination, what is the main reason? [N (%)]^**i**^	Lack of clear information Pediatricians advise	59 (10.6) 146 (26.3)
	Fear of vaccine side effects	90 (16.2)
	Increased cost of vaccines/Medical examination	91 (16.4)
	Long distance from the vaccination site	21 (3.8)
	Other^*****k*****^	86 (15.5)
	Combination of above reasons	62 (11.2)
Have you been vaccinated during your pregnancy? [N (%)]^**j**^	No Yes	1,423 (75.7) 456 (24.3)

SD, standard deviation; IQR, interquartile range;

^a^N = 1,882;

^b^N = 2,950 (total number of children who were reported by their mothers);

^c^N = 1,885;

^d^N = 1,812;

^e^N = 1,881;

^f^N = 1,854;

^g^N = 1,877;

^h^N = 1,876;

^i^N = 555;

^j^N = 1,879;

^k^Lack of vaccine/vaccination was not allowed due to illness or medication/negligence/workload.

### Vaccination attitudes and perceptions

**Figure 1:**
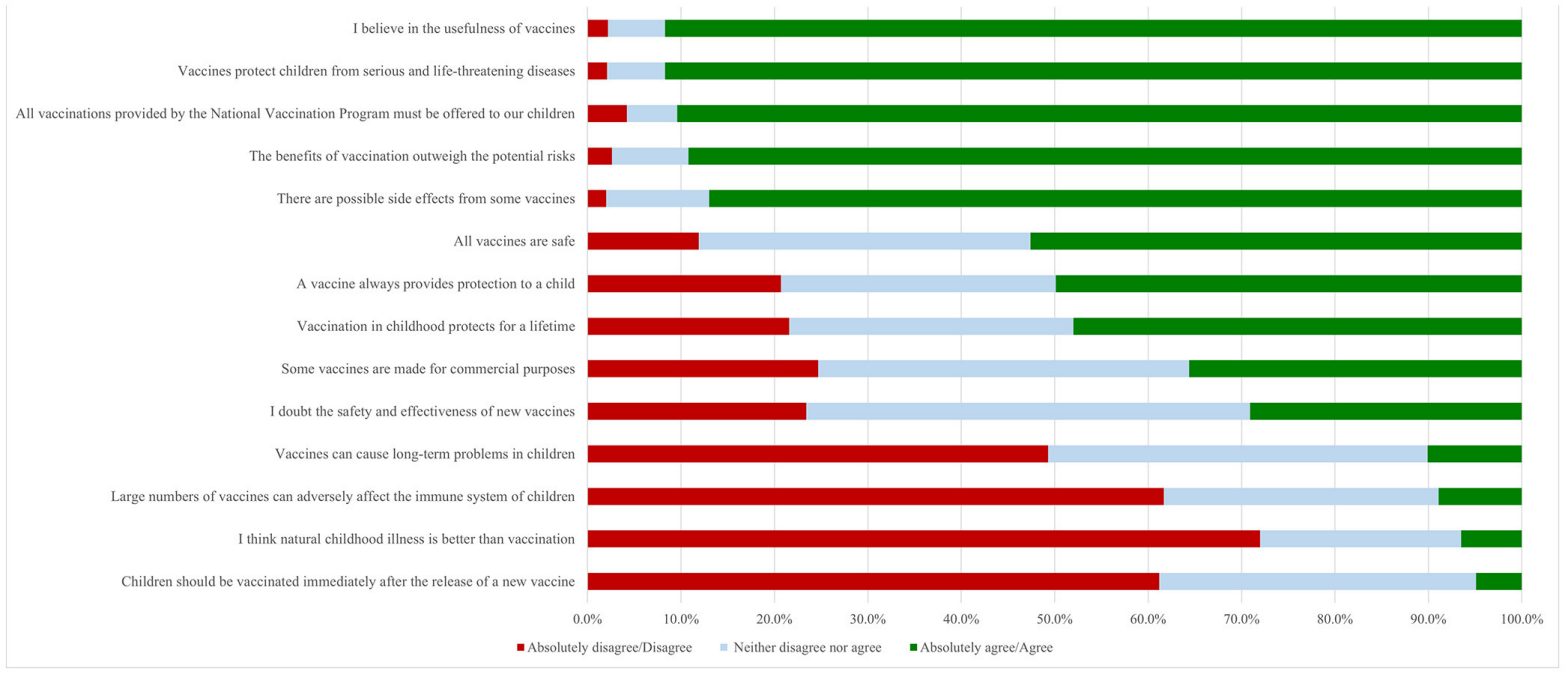
Maternal responses to questions about vaccination attitudes and perceptions.

Figure 1 presents the maternal attitudes and perceptions toward their children's vaccination. Most of the participants agreed that vaccines protect children from serious and life-threatening diseases, they believe in the usefulness of vaccines (91.7% for both statements), and they also agreed that all vaccinations provided by the National Vaccination Program must be offered to their children (90.4%). Almost half of them agreed that vaccination in childhood protects for a lifetime (48.0%) and a vaccine always provides protection to a child (49.9%). On the other hand, nearly 62% of the participants, disagreed that many vaccines can adversely affect the immune system of children (61.7%) and they should be vaccinated immediately after the release of a new vaccine (61.2%). Most of the participants did not believe that natural childhood illness is better than vaccination (72.0%). Furthermore, approximately half of mothers were neutral to the question related to the safety and effectiveness of new vaccines (47.5%).

### Vaccination attitudes and perceptions by participants' characteristics

#### Marital status, educational attainment, and single-parent status

Educational level was a significant sociodemographic factor for many of the statements. Specifically, a larger percentage of mothers with higher educational attainment agreed/absolutely agreed that all vaccinations provided by the National Vaccination Program must be offered to our children (***n*** = 1,268, 91.6%) (***p*** = 0.02) and that vaccines protect children from serious and life-threatening diseases (***n*** = 1,284, 92.9%) (***p*** = 0.01). In addition, mothers with higher education were also more likely than those with other education levels, to disagree that a large number of vaccines can adversely affect the immune system of children (***n*** = 886, 64.1%, ***p*** = 0.01). Marital status and single-parent status were significant sociodemographic predictors for one attitude. Particularly, marginally significant higher percentages of married/in cohabitation and not single-parent mothers agreed that vaccines are safe (***p*** = 0.01) (53.5 and 53.4%, respectively). In addition, a higher percentage of married/in cohabitation mothers believed in the usefulness of vaccines compared to unmarried and divorced/separated/widowed mothers (92.1 vs. 82.1% and 84.4%, respectively) (***p*** = 0.04) (Table 2).

**Table 2:** Maternal responses to questions about vaccination attitudes by marital status, educational attainment, and single parent status.

	**Marital status of mother**				**Educational attainment of mother**					**Single-parent status**				
	**Total**	**Unmarried**	**Married/** **in cohabitation**	**Divorced/** **separated/** **widowed**	***p*-value**	**Total**	**Primary**	**Secondary**	**Higher**	***p*-value**	**Total**	**No**	**Yes**	***p*-value**
Q1. All vaccinations provided by the National Vaccination Program must be offered to our children.														
D	80 (4.3)	2 (7.1)	75 (4.2)	3 (4.7)	0.05	80 (4.3)	1 (10.0)	26 (5.3)	53 (3.8)	**0.02**	79 (4.2)	72 (4.0)	7 (7.2)	0.06
N	102 (5.4)	4 (14.3)	91 (5.1)	7 (10.9)		102 (5.4)	0	39 (8.0)	63 (4.6)		101 (5.4)	92 (5.2)	9 (9.3)	
A	1,699 (90.3)	22 (78.6)	1,623 (90.7)	54 (84.4)		1,700 (90.3)	9 (90.0)	423 (86.7)	1,268 (91.6)		1,702 (90.4)	1,621 (90.8)	81 (83.5)	
Q2. All vaccines are safe.														
D	224 (11.9)	6 (21.4)	209 (11.7)	9 (14.1)	**0.01**	224 (11.9)	3 (30.0)	58 (11.9)	163 (11.8)	<0.01	223 (11.9)	208 (11.7)	15 (15.5)	**0.01**
N	668 (35.5)	13 (46.5)	623 (34.8)	32 (50.0)		668 (35.5)	4 (40.0)	216 (44.3)	448 (32.4)		668 (35.5)	623 (34.9)	45 (46.4)	
A	988 (52.6)	9 (32.1)	956 (53.5)	23 (35.9)		990 (52.6)	3 (30.0)	214 (43.8)	773 (55.8)		990 (52.6)	953 (53.4)	37 (38.1)	
Q3. Vaccines protect children from serious and life-threatening diseases.														
D	40 (2.1)	2 (7.1)	38 (2.1)	0	0.05	40 (2.1)	1 (10.0)	13 (2.7)	26 (1.9)	**0.01**	39 (2.1)	36 (2.0)	3 (3.1)	0.06
N	116 (6.2)	4 (14.3)	106 (5.9)	6 (9.4)		116 (6.2)	0	44 (9.0)	72 (5.2)		115 (6.1)	104 (5.8)	11 (11.3)	
A	1,723 (91.7)	22 (78.6)	1,643 (92.0)	58 (90.6)		1,724 (91.7)	9 (90.0)	431 (88.3)	1,284 (92.9)		1,726 (91.8)	1,643 (92.2)	83 (85.6)	
Q4. Vaccination in childhood protects for a lifetime.														
D	406 (21.6)	9 (33.3)	383 (21.4)	14 (21.9)	0.38	406 (21.6)	1 (10.0)	103 (21.1)	302 (21.9)	0.54	404 (21.5)	380 (21.3)	24 (25.0)	0.42
N	571 (30.4)	9 (33.3)	539 (30.2)	23 (35.9)		571 (30.4)	3 (30.0)	161 (33.0)	407 (29.4)		572 (30.4)	540 (30.3)	32 (33.3)	
A	902 (48.0)	9 (33.4)	866 (48.4)	27 (42.2)		903 (48.0)	6 (60.0)	224 (45.9)	673 (48.7)		904 (48.1)	864 (48.4)	40 (41.7)	
Q5. A vaccine always provides protection to a child.														
D	389 (20.7)	7 (25.0)	372 (20.8)	10 (15.6)	0.15	390 (20.7)	1 (10.0)	91 (18.7)	298 (21.6)	0.11	388 (20.6)	369 (20.7)	19 (19.6)	0.85
N	554 (29.5)	13 (46.4)	522 (29.2)	19 (29.7)		554 (29.5)	3 (30.0)	166 (34.0)	385 (27.9)		553 (29.4)	522 (29.3)	31 (32.0)	
A	936 (49.8)	8 (28.6)	893 (50.0)	35 (54.7)		936 (49.8)	6 (60.0)	231 (47.3)	699 (50.5)		939 (50.0)	892 (50.0)	47 (48.4)	
Q6. There are possible side effects from some vaccines.														
D	37 (2.0)	0	36 (2.0)	1 (1.6)	0.96	37 (2.0)	0	7 (1.4)	30 (2.2)	0.21	37 (2.0)	35 (2.0)	2 (2.1)	0.99
N	207 (11.0)	3 (10.7)	197 (11.0)	7 (10.9)		206 (10.9)	2 (20.0)	65 (13.3)	139 (10.0)		208 (11.0)	197 (11.0)	11 (11.3)	
A	1,637 (87.0)	25 (89.3)	1,556 (87.0)	56 (87.5)		1,639 (87.1)	8 (80.0)	416 (85.3)	1,215 (87.8)		1,637 (87.0)	1,553 (87.0)	84 (86.6)	
Q7. Vaccines can cause long-term problems in children.														
D	926 (49.2)	11 (39.3)	886 (49.5)	29 (45.3)	0.15	926 (49.2)	1 (10.0)	212 (43.4)	713 (51.5)	<0.01	929 (49.4)	884 (49.5)	45 (46.4)	0.19
N	764 (40.6)	13 (46.4)	728 (40.7)	23 (35.9)		765 (40.7)	8 (80.0)	218 (44.7)	539 (39.0)		764 (40.6)	727 (40.7)	37 (38.1)	
**A**	191 (10.2)	4 (14.3)	175 (9.8)	12 (18.8)		191 (10.1)	1 (10.0)	58 (11.9)	132 (9.5)		189 (10.0)	174 (9.8)	15 (15.5)	
Q8. The benefits of vaccination outweigh the potential risks.														
D	49 (2.6)	2 (7.2)	45 (2.5)	2 (3.2)	0.50	49 (2.6)	2 (20.0)	13 (2.7)	34 (2.5)	<0.01	48 (2.5)	43 (2.4)	5 (5.2)	0.11
N	155 (8.2)	3 (10.7)	145 (8.1)	7 (10.9)		155 (8.2)	0	61 (12.5)	94 (6.8)		154 (8.2)	143 (8.0)	11 (11.3)	
A	1,676 (89.2)	23 (82.1)	1,598 (89.4)	55 (85.9)		1,677 (89.2)	8 (80.0)	414 (84.8)	1,255 (90.7)		1,679 (89.3)	1,598 (89.6)	81 (83.5)	
Q9. Large number of vaccines can adversely affect the immune system of children.														
D	1,158 (61.7)	14 (50.0)	1,106 (61.9)	38 (59.4)	0.39	1,158 (61.6)	4 (40.0)	268 (55.1)	886 (64.1)	**0.01**	1,159 (61.7)	1,103 (61.9)	56 (57.7)	0.26
N	552 (29.4)	10 (35.7)	525 (29.4)	17 (26.5)		553 (29.5)	5 (50.0)	166 (34.2)	382 (27.6)		554 (29.5)	526 (29.5)	28 (28.9)	
A	168 (8.9)	4 (14.3)	155 (8.7)	9 (14.1)		168 (8.9)	1 (10.0)	52 (10.7)	115 (8.3)		166 (8.8)	153 (8.6)	13 (13.4)	
Q10. Children should be vaccinated immediately after the release of a new vaccine.														
D	1,148 (61.2)	20 (71.4)	1,087 (60.9)	41 (64.1)	0.60	1,150 (61.2)	6 (60.0)	309 (63.4)	835 (60.5)	0.38	1,148 (61.1)	1,087 (61.0)	61 (62.9)	0.73
N	636 (33.9)	6 (21.4)	611 (34.2)	19 (29.7)		635 (33.8)	4 (40.0)	161 (33.1)	470 (34.0)		637 (33.9)	607 (34.1)	30 (30.9)	
A	93 (4.9)	2 (7.2)	87 (4.9)	4 (6.3)		93 (5.0)	0	17 (3.5)	76 (5.5)		93 (5.0)	87 (4.9)	6 (6.2)	
Q11. I doubt the safety and effectiveness of new vaccines.														
D	441 (23.5)	7 (25.0)	421 (23.6)	13 (20.3)	0.39	441 (23.4)	1 (10.0)	71 (14.6)	369 (26.7)	<0.01	441 (23.4)	421 (23.6)	20 (20.6)	0.40
N	892 (47.4)	11 (39.3)	855 (47.8)	26 (40.6)		892 (47.4)	2 (20.0)	232 (47.5)	658 (47.6)		894 (47.5)	851 (47.7)	43 (44.3)	
A	547 (29.1)	10 (35.7)	512 (28.6)	25 (39.1)		548 (29.2)	7 (70.0)	185 (37.9)	356 (25.7)		546 (29.1)	512 (28.7)	34 (35.1)	
Q12. I believe in the usefulness of vaccines.														
D	41 (2.2)	2 (7.1)	37 (2.1)	2 (3.1)	**0.04**	41 (2.2)	1 (10.0)	11 (2.3)	29 (2.1)	<0.01	39 (2.1)	32 (1.8)	7 (7.2)	<0.01
N	114 (6.1)	3 (10.7)	103 (5.8)	8 (12.5)		114 (6.1)	2 (20.0)	50 (10.2)	62 (4.5)		114 (6.1)	105 (5.9)	9 (9.3)	
A	1,724 (91.7)	23 (82.1)	1,647 (92.1)	54 (84.4)		1,725 (91.7)	7 (70.0)	427 (87.5)	1,291 (93.4)		1,727 (91.8)	1,646 (92.3)	81 (83.5)	
Q13. Some vaccines are made for commercial purposes.														
D	464 (24.7)	2 (7.1)	447 (25.0)	15 (23.4)	0.20	464 (24.7)	0	93 (19.1)	371 (26.8)	<0.01	465 (24.7)	445 (25.0)	20 (20.6)	0.58
N	745 (39.6)	14 (50.0)	709 (39.7)	22 (34.4)		745 (39.6)	5 (50.0)	178 (36.5)	562 (40.7)		746 (39.7)	707 (39.6)	39 (40.2)	
A	670 (35.7)	12 (42.9)	631 (35.3)	27 (42.2)		671 (35.7)	5 (50.0)	217 (44.4)	449 (32.5)		669 (35.6)	631 (35.4)	38 (39.2)	
Q14. I think natural childhood illness is better than vaccination.														
D	1,353 (72.0)	14 (51.9)	1,291 (72.2)	48 (75.0)	0.22	1,353 (72.0)	5 (50.0)	312 (64.2)	1,036 (74.9)	<0.01	1,355 (72.1)	1,288 (72.2)	67 (69.8)	0.86
N	402 (21.4)	10 (37.0)	380 (21.3)	12 (18.8)		403 (21.4)	3 (30.0)	133 (27.4)	267 (19.3)		403 (21.5)	381 (21.4)	22 (22.9)	
A	123 (6.6)	3 (11.1)	116 (6.5)	4 (6.2)		123 (6.6)	2 (20.0)	41 (8.4)	80 (5.8)		121 (6.4)	114 (6.4)	7 (7.3)	

D, absolutely disagree/disagree; N, neither disagree nor agree; A, agree/absolutely agree; Bold font indicates statistical significance after a Bonferroni correction (p < 0.05).

#### Employment and income status

A considerably higher proportion of mothers with high income (***n*** = 547, 52.1%) agreed that vaccine always provides protection to a child compared to mothers without income (***n*** = 20, 41.7%) (***p*** = 0.03). In addition, a significant higher percentage of mothers with low, middle, or high income and employed mothers agreed that some vaccines have possible side effects compared to mothers without income (***p*** = 0.04) and unemployed mothers (***p*** = 0.01) (Table 3). Moreover, a significantly lower percentage of private employees (***n*** = 56, 7.6%) (***p*** = 0.03) and participants with a low income (***n*** = 24, 8.7%) (***p*** = 0.02) agreed that vaccines can cause long-term problems in children were identified compared to the other employment and income categories, respectively. A smaller percentage of unemployed mothers disagreed that some vaccines are made for commercial purposes (***n*** = 84, 18.1%) (***p*** = 0.02) and natural childhood illness is better than vaccination (***n*** = 311, 67.3%) (***p*** = 0.01) compared to employed participants. We also found that mothers without income disagreed in a significantly higher proportion, compared to the other categories, that children should be vaccinated immediately after the release of a new vaccine (***n*** = 38, 79.2%) (***p*** = 0.01). In the same sense, a significantly lower percentage of participants without income disagreed that large numbers of vaccines can adversely affect the immune system of children (***n*** = 20, 41.7%) (***p*** = 0.02). More information about the maternal responses to questions about vaccination attitudes by employment and income status of the mother is presented in Table 3.

**Table 3:** Maternal responses to questions about vaccination attitudes by employment and income status of the mother.

	**Employment status of mother**					**Income status of mother**					
	**Total**	**Unemployed**	**State employee**	**Private employee**	**Freelance**	***p*-value**	**Total**	**None**	**Low**	**Moderate**	**High**	***p*-value**
Q1. All vaccinations provided by the National Vaccination Program must be offered to our children.												
D	79 (4.2)	22 (4.7)	16 (4.6)	23 (3.1)	18 (5.4)	0.15	79 (4.3)	4 (8.3)	9 (3.3)	20 (4.2)	46 (4.4)	0.27
N	102 (5.4)	23 (5.0)	19 (5.4)	34 (4.6)	26 (7.9)		99 (5.3)	5 (10.4)	12 (4.4)	31 (6.4)	51 (4.9)	
A	1,701 (90.4)	418 (90.3)	314 (90.0)	683 (92.3)	286 (86.7)		1,676 (90.4)	39 (81.3)	255 (92.3)	430 (89.4)	952 (90.7)	
Q2. All vaccines are safe.												
D	223 (11.9)	60 (13.0)	50 (14.3)	60 (8.1)	53 (16.1)	<0.01	220 (11.9)	11 (22.9)	23 (8.3)	61 (12.6)	125 (12.0)	<0.01
N	667 (35.4)	174 (37.6)	104 (29.8)	281 (38.0)	108 (32.7)		656 (35.4)	17 (35.4)	127 (46.0)	171 (35.6)	341 (32.5)	
A	991 (52.7)	229 (49.4)	195 (55.9)	398 (53.9)	169 (51.2)		977 (52.7)	20 (41.7)	126 (45.7)	249 (51.8)	582 (55.5)	
Q3. Vaccines protect children from serious and life-threatening diseases.												
D	39 (2.1)	12 (2.6)	7 (2.0)	13 (1.8)	7 (2.1)	0.58	39 (2.1)	2 (4.2)	5 (1.8)	13 (2.7)	19 (1.8)	<0.01
N	116 (6.2)	34 (7.3)	21 (6.0)	37 (5.0)	24 (7.3)		113 (6.1)	10 (20.8)	18 (6.5)	33 (6.9)	52 (5.0)	
A	1,725 (91.7)	417 (90.1)	320 (92.0)	690 (93.2)	298 (90.6)		1,700 (91.8)	36 (75.0)	253 (91.7)	434 (90.4)	977 (93.2)	
Q4. Vaccination in childhood protects for a lifetime.												
D	405 (21.5)	96 (20.8)	88 (25.3)	145 (19.6)	76 (23.0)	0.30	398 (21.5)	10 (20.8)	44 (15.9)	106 (22.0)	238 (22.7)	0.09
N	572 (30.4)	149 (32.2)	107 (30.7)	222 (30.0)	94 (28.5)		564 (30.4)	20 (41.7)	90 (32.6)	154 (32.0)	300 (28.6)	
A	903 (48.1)	217 (47.0)	153 (44.0)	373 (50.4)	160 (48.5)		891 (48.1)	18 (37.5)	142 (51.5)	221 (46.0)	510 (48.7)	
Q5. A vaccine always provides protection to a child.												
D	389 (20.7)	95 (20.6)	75 (21.5)	147 (19.9)	72 (21.9)	0.76	384 (20.7)	13 (27.1)	43 (15.6)	107 (22.3)	221 (21.1)	**0.03**
N	553 (29.4)	147 (31.8)	102 (29.2)	207 (28.0)	97 (29.5)		543 (29.3)	15 (31.2)	96 (34.9)	151 (31.5)	281 (26.8)	
A	938 (49.9)	220 (47.6)	172 (49.3)	386 (52.2)	160 (48.6)		925 (50.0)	20 (41.7)	136 (49.5)	222 (46.2)	547 (52.1)	
Q6. There are possible side effects from some vaccines.												
D	37 (2.0)	6 (1.3)	6 (1.7)	12 (1.6)	13 (3.9)	**0.01**	37 (2.0)	2 (4.2)	2 (0.7)	6 (1.3)	27 (2.6)	**0.04**
N	207 (11.0)	63 (13.6)	34 (9.8)	87 (11.8)	23 (7.0)		203 (10.9)	9 (18.7)	38 (13.8)	53 (11.0)	103 (9.8)	
A	1,638 (87.0)	394 (85.1)	309 (88.5)	641 (86.6)	294 (89.1)		1,614 (87.1)	37 (77.1)	236 (85.5)	422 (87.7)	919 (87.6)	
Q7. Vaccines can cause long-term problems in children.												
D	928 (49.3)	212 (45.8)	176 (50.4)	373 (50.4)	167 (50.6)	**0.03**	914 (49.3)	21 (43.8)	130 (47.1)	215 (44.7)	548 (52.2)	**0.02**
N	764 (40.6)	202 (43.6)	132 (37.8)	311 (42.0)	119 (36.1)		752 (40.6)	18 (37.5)	122 (44.2)	218 (45.3)	394 (37.6)	
A	190 (10.1)	49 (10.6)	41 (11.8)	56 (7.6)	44 (13.3)		188 (10.1)	9 (18.7)	24 (8.7)	48 (10.0)	107 (10.2)	
Q8. The benefits of vaccination outweigh the potential risks.												
D	48 (2.6)	12 (2.6)	9 (2.6)	13 (1.8)	14 (4.3)	0.11	48 (2.6)	2 (4.2)	5 (1.8)	13 (2.7)	28 (2.7)	<0.01
N	155 (8.2)	45 (9.7)	32 (9.2)	49 (6.6)	29 (8.8)		152 (8.2)	13 (27.1)	28 (10.2)	42 (8.7)	69 (6.6)	
A	1,678 (89.2)	406 (87.7)	308 (88.2)	678 (91.6)	286 (86.9)		1,653 (89.2)	33 (68.7)	243 (88.0)	425 (88.6)	952 (90.7)	
Q9. Large number of vaccines can adversely affect the immune system of children.												
D	1,159 (61.7)	272 (58.7)	220 (63.0)	466 (63.1)	201 (61.1)	<0.01	1,145 (61.9)	20 (41.7)	160 (58.2)	297 (61.8)	668 (63.8)	**0.02**
N	553 (29.4)	143 (30.9)	99 (28.4)	228 (30.9)	83 (25.2)		542 (29.2)	21 (43.7)	96 (34.9)	141 (29.3)	284 (27.1)	
A	167 (8.9)	48 (10.4)	30 (8.6)	44 (6.0)	45 (13.7)		164 (8.9)	7 (14.6)	19 (6.9)	43 (8.9)	95 (9.1)	
Q10. Children should be vaccinated immediately after the release of a new vaccine.												
D	1,148 (61.1)	304 (65.7)	208 (59.9)	440 (59.5)	196 (59.6)	0.06	1,135 (61.3)	38 (79.2)	189 (68.5)	292 (60.8)	616 (58.8)	**0.01**
N	637 (33.9)	148 (31.9)	117 (33.7)	260 (35.2)	112 (34.0)		625 (33.8)	7 (14.6)	76 (27.5)	168 (35.0)	374 (35.7)	
A	93 (5.0)	11 (2.4)	22 (6.4)	39 (5.3)	21 (6.4)		91 (4.9)	3 (6.2)	11 (4.0)	20 (4.2)	57 (5.4)	
Q11. I doubt the safety and effectiveness of new vaccines.												
D	441 (23.4)	76 (16.4)	94 (27.0)	179 (24.2)	92 (27.9)	<0.01	434 (23.4)	7 (14.6)	51 (18.5)	101 (21.0)	275 (26.2)	**0.01**
N	895 (47.6)	222 (48.0)	163 (46.8)	364 (49.2)	146 (44.2)		879 (47.4)	21 (43.7)	131 (47.5)	227 (47.2)	500 (47.7)	
A	545 (29.0)	165 (35.6)	91 (26.2)	197 (26.6)	92 (27.9)		541 (29.2)	20 (41.7)	94 (34.0)	153 (31.8)	274 (26.1)	
Q12. I believe in the usefulness of vaccines.												
D	40 (2.1)	9 (1.9)	9 (2.6)	12 (1.6)	10 (3.0)	0.59	41 (2.2)	3 (6.3)	6 (2.2)	8 (1.7)	24 (2.3)	<0.01
N	114 (6.1)	31 (6.7)	22 (6.3)	38 (5.2)	23 (7.0)		112 (6.1)	11 (22.9)	19 (6.9)	30 (6.2)	52 (5.0)	
A	1,726 (91.8)	423 (91.4)	318 (91.1)	688 (93.2)	297 (90.0)		1,699 (91.7)	34 (70.8)	251 (90.9)	442 (92.1)	972 (92.7)	
Q13. Some vaccines are made for commercial purposes.												
D	465 (24.7)	84 (18.1)	89 (25.7)	201 (27.2)	91 (27.5)	**0.02**	459 (24.8)	7 (14.6)	37 (13.4)	120 (25.0)	295 (28.1)	<0.01
N	746 (39.7)	199 (43.0)	134 (38.6)	292 (39.5)	121 (36.7)		733 (39.5)	13 (27.1)	129 (46.7)	183 (38.1)	408 (38.9)	
A	669 (35.6)	180 (38.9)	124 (35.7)	247 (33.3)	118 (35.8)		661 (35.7)	28 (58.3)	110 (39.9)	177 (36.9)	346 (33.0)	
Q14. I think natural childhood illness is better than vaccination.												
D	1,354 (72.1)	311 (67.3)	244 (69.9)	553 (74.8)	246 (74.8)	**0.01**	1,333 (72.0)	24 (50.0)	186 (67.4)	339 (70.8)	784 (74.8)	<0.01
N	403 (21.4)	121 (26.2)	78 (22.4)	149 (20.2)	55 (16.7)		398 (21.5)	18 (37.5)	81 (29.3)	107 (22.3)	192 (18.3)	
A	122 (6.5)	30 (6.5)	27 (7.7)	37 (5.0)	28 (8.5)		120 (6.5)	6 (12.5)	9 (3.3)	33 (6.9)	72 (6.9)	

D, absolutely disagree/disagree; N, neither disagree nor agree; A, agree/absolutely agree; Bold font indicates statistical significance after a Bonferroni correction (p < 0.05).

#### Age of mothers and children

Regarding mothers' age, significant differences are observed in the statements “a vaccine always provides protection to a child” (***p*** < 0.01), “there are possible side effects from some vaccines” (***p*** = 0.01), “I doubt the safety and effectiveness of new vaccines” (***p*** = 0.02), and “some vaccines are made for commercial purposes” (***p*** = 0.02). The age of the children noted significant differences only for the attitude “I doubt the safety and effectiveness of new vaccines” (***p*** = 0.04) (Supplementary Table 1).

#### Geographical region and area of residency

Region of residency was a significant sociodemographic factor for the statements “vaccines can cause long-term problems in children” and “some vaccines are made for commercial purposes” (Supplementary Table 2). More specifically, 53.7% (***n*** = 474) of the Attica residents disagreed that vaccines could cause long-term problems, while significantly lower (***p*** = 0.04) was the corresponding percentage for the residents of Central (***n*** = 112, 45.7%) and North Greece (***n*** = 247, 45.2%), as well as Crete and Aegean Islands (***n*** = 96, 45.5%). In addition, almost half of the mothers who lived in Crete and Aegean Islands (***n*** = 100, 47.4%), remained neutral regarding the commercial purposes of vaccines (***p*** = 0.02). This attitude was significantly lower in the other geographical areas of Greece, while 33.6% (***n*** = 71) of the remaining residents of Crete and Aegean Island agreed with this statement. Furthermore, in rural areas, a significantly higher proportion of participants, disagreed that children should be vaccinated immediately after the release of a new vaccine (67.2 vs. 60.4% in urban areas, ***p*** = 0.03).

#### Previous vaccination behavior

Several statistically significant associations were observed between maternal attitudes toward childhood vaccination and maternal vaccination behaviors. The majority of those who adhere to the prescribed dosage as indicated by the local recommendations for each vaccine, delayed their children's vaccination, and were vaccinated during pregnancy, agreed that all the vaccinations provided by the National Vaccination Program must be offered to their children, all vaccines are safe, and vaccines protect children from serious and life-threatening diseases (all *p* < 0.05). In addition, most mothers who adhered to the prescribed dosage as indicated by the local recommendations for each vaccine and those who did not delay their children vaccination agreed that vaccination in childhood protects for a lifetime and a vaccine always provides protection to a child (*p* < 0.01). Finally, significant associations were noted between all previous maternal vaccination behaviors and attitudes toward vaccines associated with long-term problems benefits, safety and efficacy, usefulness, commercial purposes, new vaccine acceptance, and preference of natural illness compared to vaccination (Supplementary Table 3).

## Discussion

To the best of our knowledge, this is the first study that evaluated the attitudes and perceptions of mothers in Greece concerning the vaccination of their children during the COVID-19 pandemic. Our findings show that most of the participants agreed that vaccines protect children from serious and life-threatening diseases, and they believe in the usefulness of vaccines. In addition, most of the participants did not believe that natural childhood illness is better than vaccination and approximately half of them were neutral to the question of whether they doubt the safety and effectiveness of new vaccines. Sociodemographic factors significantly affected the maternal attitudes and perceptions toward childhood vaccination. About half of the participants with low income, secondary and higher education, reported neutral attitudes. In Aegean Islands and Crete, a significantly higher prevalence of negative and neutral vaccination perceptions was observed, while negative attitudes were reported in both rural and urban areas. The age of mothers influences their attitudes and predictions about childhood vaccination. Finally, several significant associations were observed between maternal attitudes toward childhood vaccination and previous maternal vaccination behaviors such as adherence to recommender vaccine dosages, vaccination delays, and vaccination uptake during pregnancy.

Statistically significant associations between sociodemographic and economic factors and maternal attitudes have been found in our research. The marital status and educational level are determinants of vaccine belief and attitudes in some countries. In our study, the unmarried, divorced, separated, or widowed mothers were less likely to associate with positive beliefs about vaccine usefulness. Divorced parents in the United Arab Emirates were more vaccine-hesitant (24), while married mothers in Nigeria were associated with commitment to childhood vaccination (56). In contrast, the marital status was not associated with positive attitudes in Croatia (57). The income and educational level were identified as important factors in our study, since primarily educated mothers and single parents without income have negative attitudes toward vaccination. In contrast to our findings, a study in Israel identified higher vaccination compliance among parents with low educational level and average wages (58), and highly educated mothers in Georgia were prone to complete all the childhood vaccinations (59). A previous cross-sectional study in Greece suggests an association between children's age and incomplete vaccination uptake (60). Our results show a statistically significant association only between children's and mothers' age and the safety and effectiveness of new vaccines. Different geographical areas in Greece were associated with different vaccination perceptions, which was also observed in some areas in Italy (61).

The impact of education and income on maternal attitudes and perceptions toward childhood vaccination was also observed in this study. Maternal attitudes toward vaccination protection, safety, efficacy, usefulness, and commercial purposes were linked to maternal educational attainment, income and/or marital status, and age. Our research is consistent with the worldwide literature, which identifies education and income as crucial determinants of vaccination uptake and knowledge across the world (62–65). Also, a previously published study in Greece identified parental age, educational level, and marital status as an important influence of vaccination coverage (66). The results of our study can be used for future campaign to target specific groups to enhance positive attitudes toward vaccination. It is crucial to plan and design effective campaigns which will be population specific according to the maternal socio-demographic characteristics.

Conspiracy theories around childhood vaccination have been previously reported to influence childhood vaccination uptake (67, 68). To date, there has been no detailed investigation of this topic in Greece, however, few studies are providing preliminary insights into parental belief in conspiracy theories. Among them, our study showed that the majority of mothers agreed or remained neutral on the question related to the commercial purpose of vaccines, while a prior study revealed that some mothers support the link between vaccines and autism (66). A recent study in Greece identified the fear of vaccine-related side effects as the main reason of childhood COVID-19 vaccination refusal, while conspiracy theories (vaccines have other purposes) was not a popular reason (69). To develop a full picture of the role of conspiracy theories on childhood vaccination uptake in Greece, additional studies will be needed.

Regarding the positive, negative, and neutral attitudes, we observed different attitudes depending on the vaccination question. Positive maternal attitudes were recorded to general questions such as the vaccination protection, usefulness, and supply through the national vaccination program. On the other hand, fewer positive attitudes were recorded when mothers were asked about the safety of all vaccines and lifetime protection. Mothers possessed neutral attitude toward specific vaccination questions such as the safety and efficacy of new vaccines and the commercial purposes of some vaccines. We identified a considerably high ratio of mothers with neutral attitudes concerning the long-term vaccine-related problems, the safety and efficacy of the new vaccine, and the commercial purposes of some vaccines. The prevalent neutral attitudes toward specific vaccination questions may change to positive attitudes after interventions. Prior studies have noted the importance of educational interventions in parental knowledge and attitudes toward the vaccination of their children (70, 71). We believe that population-specific educational interventions in the Greek cohort are necessary to maintain a positive attitude toward vaccination and prevent a high level of vaccination hesitancy and negative attitudes toward vaccination in the future.

There are several important differences and similarities between our research and other studies in Europe. Most of the participants in our study did not believe that natural childhood illness is better than vaccination. By contrast, many parents in Finland supported that natural illness provides longer protection compared to vaccination (72). Interestingly, we revealed that a high proportion of women in Greece have not been vaccinated during pregnancy. Similarly, only a small percentage of women in Italy and Germany have received vaccinations during pregnancy (73, 74), while the uptake in the UK was considerably higher (75). Of note, women in the UK stated that pregnancy interferes with their decision to accept the COVID-19 vaccine (76). In regard to childhood vaccination delays, those were also observed in other European countries such as France, Belgium, and Albania (77–79).

It is also important to acknowledge the role of healthcare professionals in reshaping maternal attitudes toward vaccination. Literature highlights the positive influence of parental trust toward pediatricians and vaccination uptake (80). Previous studies among mothers and pregnant women in Greece and in Cyprus identified pediatricians as the primary and trustworthy source of vaccination-related information (55, 81, 82). In addition, two of those studies revealed the influence of pediatricians on childhood vaccination delay (55, 82). Therefore, interventions through pediatricians in Greece seem to be effective. Pediatricians should inform mothers, especially those with characteristics linked to negative and neutral attitudes, about vaccination benefits. A cost-effective approach is the development of a free mobile application with videos about vaccination which will be promoted by pediatricians (83, 84).

The prevalent negative and neutral attitudes toward vaccination may lead to inadequate vaccination uptake (65), and influence new vaccine acceptance (85). Negative maternal attitudes toward new vaccines were previously shown in the Greek cohort (60). In our study, most mothers were neutral regarding the safety and efficacy of new vaccines and opposed to the immediate vaccination after a new vaccine release. The low levels of new vaccine acceptance found in our study raise concerns about the acceptability of future vaccines such as the COVID-19 vaccine. Future studies focusing on new vaccine trust among mothers in Greece are therefore recommended. Nevertheless, the majority recognized the vaccine's usefulness and disease prevention and agreed that the benefits outweigh any potential vaccination risks. These findings raise intriguing questions regarding the process of developing trust and confidence toward new vaccines.

Our study also revealed that parental vaccination behaviors are closely related to their attitudes toward childhood vaccination. Mothers who adhered to the recommended dosages for each childhood vaccine did not delay the vaccination of their children and have been vaccinated during pregnancy have shown more positive attitudes compared to those who did not adhere to the prescribed vaccine dosages, delayed vaccination, and did not receive vaccinations during pregnancy. Research in the past few years also acknowledges that parental vaccination behavior and experiences influence childhood vaccination uptake, especially that vaccination-related side effects can cause delays or vaccine refusal (51). In addition, acceptance of seasonal influenza vaccination during pregnancy was associated with maternal intention to vaccinate their infants (86).

The COVID-19 pandemic has initiated conversations around regulations to minimize the transmission of the novel coronavirus while preserving human rights and diminishing health inequalities. COVID-19 vaccine approval and availability are milestones in the history of humanity and highlighted the emergency of understanding the vaccine hesitancy. Our findings may help us to understand the parental attitudes toward the childhood COVID-19 vaccination, since reveals the maternal sociodemographic characteristics and how they influence their attitudes and perceptions toward childhood vaccination at the beginning of the pandemic. Previous studies have shown that parental confidence in the COVID-19 vaccine positively influenced the vaccine acceptance for their children (87), while positive attitudes toward childhood vaccination and belief in the importance of vaccines do not reflect their willingness to accept the COVID-19 vaccination of their children (88). Here, we identified a high level of maternal hesitancy toward new vaccines, and specific maternal characteristics were associated with negative attitudes and perceptions. Although a higher percentage of mothers who received vaccinations during pregnancy agreed that children should be vaccinated immediately after the release of new vaccines, the majority remained neutral. It is crucial to provide useful sources of vaccination-related information, with a focus on topics such as vaccine development and approval to those groups and implement strategies to minimize the parental hesitancy toward childhood vaccination and increase the acceptance of new vaccines.

Despite our major research findings, this study has some limitations. Firstly, the data collection was done using convenience sampling through an online tool that limits our study representativeness. Our sample includes a higher proportion of highly educated women compared to the general population as well as a higher proportion of women living in urban areas, which could lead to selection bias. Nevertheless, the overrepresentation of such characteristics is possible to reflect greater health awareness and interest in science, whilst the use of online methods is the best solution for data collection in periods of social distancing due to the COVID-19 pandemic. Secondly, the self-reporting nature of data collection potentially leads to misreporting and information bias and potential under-or overestimations of reported associations. However, the latter is less of an issue as it is inherent in all types of knowledge/attitude assessment research. Thirdly, this is a cross-sectional study, therefore causal relationships between mothers' knowledge and vaccination behavior cannot be inferred. Despite these limitations, our study involved a fair number of participants with different social-demographic characteristics at a national level, however, the generalizability of the findings may be limited by possible selection bias due to language barrier, unfamiliarity with online survey tools, and the oversampling of a particular network of similar groups.

## Conclusions

The study provides knowledge about the attitudes and perceptions of mothers in Greece regarding childhood vaccination. Most of the mothers participated in the study believe in the usefulness of vaccines and that vaccines protect children from serious and life-threatening diseases. In addition, the majority of the mothers strictly adhered to the prescribed dosage as indicated by the local recommendations for each vaccine, have delayed their child/children vaccination and have not been vaccinated during pregnancy. Educational level was a significant sociodemographic factor for most many of the attitudes. In addition, maternal attitudes toward childhood vaccination and previous maternal practices (i.e., adherence to recommender vaccine dosages, vaccination delays, and vaccination uptake during pregnancy) were associated with attitudes and perceptions about vaccination. Given the fact that multiple epidemics, outbreaks, and pandemics are happening worldwide over the years, revealing the attitudes and perceptions of mothers about childhood vaccination is essential for public health planning. The neutral attitudes toward vaccination raise concerns about possible negative attitudes in the future, while the previous parental vaccination behavior affects the childhood vaccination outcome. Healthcare professionals and public health decision-makers should consider the attitudes and perceptions of mothers on the development of educational programs and campaigns, by informing and encouraging mothers about childhood vaccination. This will be critical in the coming years, as mothers' vaccination practices for their children are anticipated to become increasingly significant in the wake of the COVID-19 pandemic.

## Data availability statement

The raw data supporting the conclusions of this article will be made available by the authors, without undue reservation.

## Ethics statement

This study was conducted according to the guidelines laid down in the Declaration of Helsinki and all procedures involving research study participants were approved by the Cyprus National Bioethics Committee (EEBK EΠ 2020.01.82). The participants provided their consent to participate in this study.

## Author contributions

GF, MK, and KG drafted the original manuscript. GF and KG interpreted the results. AH, MK, and KG analyzed the data. ET, MK, and KG designed the survey and collected data. KG conceived and supervised this research. All authors read and approved the final manuscript.

## Conflict of interest

The authors declare that the research was conducted in the absence of any commercial or financial relationships that could be construed as a potential conflict of interest.

## Publisher's note

All claims expressed in this article are solely those of the authors and do not necessarily represent those of their affiliated organizations, or those of the publisher, the editors and the reviewers. Any product that may be evaluated in this article, or claim that may be made by its manufacturer, is not guaranteed or endorsed by the publisher.
