# Supplementary material for: Attitudes and perceptions of mothers towards childhood vaccination in Greece: lessons to improve the childhood COVID-19 vaccination acceptance
Source: Front Pediatr. 2022 Aug 25;10:951039. doi: 10.3389/fped.2022.951039 (PMC9453258; doi:10.3389/fped.2022.951039)
Supplement: Supplementary file 1 [file Data_Sheet_1.pdf]

Supplementary file: Electronic consent form and questionnaire in Greek and translated to English. (The original form in Greek is provided below).

ATTITUDES, KNOWLEDGE, AND PERCEPTIONS OF PARENTS TOWARDS VACCINATION

What is the purpose of this research?

The purpose of this questionnaire is the investigation of parents' attitudes, knowledge, and perceptions regarding the vaccination of their children. The questionnaire is anonymous, and your information will remain within the scope of the survey, without the risk of using it for another purpose. The purpose of the research is purely scientific. The results of the research can be compared with similar studies and will contribute to the promotion of public health. The study is aimed at parents or guardians with at least one minor child (<18 years old).

Important information

You will be asked to answer a short anonymous questionnaire. Your participation is anonymous and voluntary, and the time required to complete the questionnaire ranges from 8 to 10 minutes. Please choose the answer that represents your point of view and answer as honestly as possible. All information you provide to us will remain strictly confidential and will not be disclosed to anyone other than the research team. If you would like to withdraw your participation, even after submitting the answers, contact 22559656 by phone to the lead study coordinator: Dr Konstantinos Giannakou (K.Giannakou@euc.ac.cy). The present research has not received any funding and has been approved by the National Bioethics Committee of Cyprus (ΕΕΒΚ ΕΠ 2020.01.82).

Do you agree to take part in the study?

- o Yes
- o No

SECTION A – SOCIO-DEMOGRAPHICS

A1. What is your gender?
- Male
- Female
- Other (Specify)

A2. What is your age?
- __________

A3. What is your country of origin?
- Cyprus
- Greece
- Other (Specify)

A4. Which city do you live in?
- Athens
- Thessaloniki
- Nicosia
- Larnaca
- Limassol
- Other (Specify)

A5. In which area do you live?
- Rural
- Urban

A6. What is your profession?
- Private employee
- State employee
- Freelance
- Unemployed
- Housewife
- Student
- Retired
- Other (Specify)

A7. Are you a health professional (e.g., doctor, nurse, etc.)?
- Yes
- No

A8. What is your marital status?
- Married
- In cohabitation
- Unmarried
- Divorced
- Separated
- Widowed

A9. What is the highest level of education you have completed so far?
- Primary school

- Gymnasium
- High School/Technical school
- College
- Bachelor/Undergraduate
- Master/Postgraduate
- Doctorate

A10. What is your religion?
- Christian Orthodox
- Christian Catholic
- Muslim
- Jehovah's Witness
- Other (Specify)

A11. What is your monthly gross income (euros)?
- No income
- Less than 1001
- 1001-1500
- 1501-2000
- More than 2001

SECTION B - INFORMATION ON VACCINATION STATUS

B1. Are you a single parent family?
- o Yes
- o No

B2. Number of children
- o __________

B3. Determine the gender of your children
- o 1st child Boy □ Girl □
- o 2nd child Boy □ Girl □
- o 3rd child Boy □ Girl □
- o 4th child Boy □ Girl □
- o 5th child Boy □ Girl □
- o 6th child Boy □ Girl □

B4. What are the ages of your children (for example, 1st child = 2 years old, 2nd child = 4 years old, etc.)?
- o __________

B5. Do you follow the prescribed doses as indicated by local recommendations for each vaccine?
- o Yes
- o No

SECTION C – GENERAL VACCINATION KNOWLEDGE

C1. Vaccines are unnecessary, as viruses can be treated with antibiotics.
- True
- False
- I do not know

C2. The effectiveness of vaccines has been demonstrated by epidemiological studies.
- True
- False
- I do not know

C3. Systematic vaccination helped to reduce or eliminate many infectious diseases worldwide.
- True
- False
- I do not know

C4. Vaccination can be done in summer.
- True
- False
- I do not know

C5. Vaccination can be done when my child has a cold.
- True
- False
- I do not know

C6. Vaccination can be done when my child has a fever (>38℃).
- True
- False
- I do not know

C7. Vaccine for measles/rubella/rubella/mumps (MMR) is associated with autism.
- True
- False
- I do not know

C8. Children would be more resistant if they were not vaccinated.
- True
- False
- I do not know

C9. Many vaccines are given too early, leaving the children's immune system, unable to develop.
- True
- False
- I do not know

C10. The doses of chemicals that are used in the vaccines are dangerous for humans.
- True
- False
- I do not know

C11. Vaccination increases the appearance of allergies.

- o True
- o False
- o I do not know

C12. There is a vaccine to prevent cervical cancer.

- o True
- o False
- o I do not know

C13. Vaccination is not needed for diseases that have disappeared.

- o True
- o False
- o I do not know

SECTION D - QUESTIONS ABOUT ATTITUDES TOWARDS VACCINATION

D1. All vaccinations provided by the National Vaccination Program must be offered to our children.
- o Strongly disagree
- o Disagree
- o Neither agree nor disagree
- o Agree
- o Strongly agree

D2. All vaccines are safe.
- o Strongly disagree
- o Disagree
- o Neither agree nor disagree
- o Agree
- o Strongly agree

D3. Vaccines protect children from serious and life-threatening diseases.
- o Strongly disagree
- o Disagree
- o Neither agree nor disagree
- o Agree
- o Strongly agree

D4. Vaccination in childhood protects for a lifetime.
- o Strongly disagree
- o Disagree
- o Neither agree nor disagree
- o Agree
- o Strongly agree

D5. A vaccine always provides protection to a child.
- o Strongly disagree
- o Disagree
- o Neither agree nor disagree
- o Agree
- o Strongly agree

D6. There are possible side effects from some vaccines.
- o Strongly disagree
- o Disagree
- o Neither agree nor disagree
- o Agree
- o Strongly agree

D7. Vaccines can cause long-term problems in children.
- o Strongly disagree
- o Disagree
- o Neither agree nor disagree
- o Agree
- o Strongly agree

D8. The benefits of vaccination outweigh the potential risks.
- Strongly disagree
- Disagree
- Neither agree nor disagree
- Agree
- Strongly agree

D9. Large number of vaccines can adversely affect the immune system of children.
- Strongly disagree
- Disagree
- Neither agree nor disagree
- Agree
- Strongly agree

D10. Children should be vaccinated immediately after the release of a new vaccine.
- Strongly disagree
- Disagree
- Neither agree nor disagree
- Agree
- Strongly agree

D11. I doubt the safety and effectiveness of new vaccines.
- Strongly disagree
- Disagree
- Neither agree nor disagree
- Agree
- Strongly agree

D12. I believe in the usefulness of vaccines.
- Strongly disagree
- Disagree
- Neither agree nor disagree
- Agree
- Strongly agree

D13. Some vaccines are made for commercial purposes.
- Strongly disagree
- Disagree
- Neither agree nor disagree
- Agree
- Strongly agree

D14. I think natural childhood illness is better than vaccination.
- Strongly disagree
- Disagree
- Neither agree nor disagree
- Agree
- Strongly agree

SECTION E. SOURCES OF INFORMATION AND GENERAL QUESTIONS ABOUT VACCINATION

E1. I am sufficiently informed about vaccination.
- Strongly disagree
- Disagree
- Neither agree nor disagree
- Agree
- Strongly agree

E2. What is the main source of information for you about vaccinating your children?
- Pediatrician
- Pharmacist
- Family Doctor
- Personal Doctor
- Internet and media
- Family and friends
- Other (Specify)

E3. I completely trust my child's pediatrician.
- Strongly disagree
- Disagree
- Neither agree nor disagree
- Agree
- Strongly agree

E4. I freely discuss my concerns with the pediatrician.
- Strongly disagree
- Disagree
- Neither agree nor disagree
- Agree
- Strongly agree

E5. Have you ever delayed your child/children vaccination?
- Yes
- No

E6. If you have delayed your child/children vaccination, what were the main reasons.
- Illness
- Lack of clear information
- Pediatrician’s suggestion
- Fear of side effects of the vaccine
- Increased cost of vaccines
- Increased cost of medical visit
- Long distance from the vaccination site
- Other (Specify)

E7. Have you vaccinated during your pregnancy?
- Yes
- No

ΣΤΑΣΕΙΣ, ΓΝΩΣΕΙΣ ΚΑΙ ΑΝΤΙΛΗΨΕΙΣ ΓΟΝΕΩΝ ΩΣ ΠΡΟΣ ΤΟΝ ΕΜΒΟΛΙΑΣΜΟ ΤΩΝ ΠΑΙΔΙΩΝ ΤΟΥΣ

Ποιος είναι ο σκοπός της έρευνας;
Σκοπός αυτού του ερωτηματολογίου είναι η διερεύνηση των στάσεων, των γνώσεων και των αντιλήψεων των γονέων σχετικά με τον εμβολιασμό των παιδιών τους. Το ερωτηματολόγιο είναι ανώνυμο και οι πληροφορίες σας θα παραμείνουν εντός του πεδίου εφαρμογής της έρευνας, χωρίς τον κίνδυνο χρήσης τους για άλλο σκοπό. Ο σκοπός της έρευνας είναι καθαρά επιστημονικός. Τα αποτελέσματα της έρευνας μπορούν να συγκριθούν με παρόμοιες μελέτες και θα συμβάλουν στην προαγωγή της δημόσιας υγείας. Η μελέτη απευθύνεται σε γονείς ή κηδεμόνες με τουλάχιστον ένα ανήλικο παιδί (<18 ετών).

Σημαντικές πληροφορίες
Θα σας ζητηθεί να απαντήσετε σε ένα σύντομο ανώνυμο ερωτηματολόγιο. Η συμμετοχή σας είναι ανώνυμη και εθελοντική και ο χρόνος που απαιτείται για τη συμπλήρωση του ερωτηματολογίου κυμαίνεται από 8 έως 10 λεπτά. Επιλέξτε την απάντηση που αντιπροσωπεύει την άποψή σας και απαντήστε όσο το δυνατόν πιο ειλικρινά. Όλες οι πληροφορίες που μας παρέχετε θα παραμείνουν αυστηρά εμπιστευτικές και δεν θα γνωστοποιηθούν σε κανέναν άλλο εκτός από την ερευνητική ομάδα. Εάν επιθυμείτε να αποσύρετε τη συμμετοχή σας, ακόμη και μετά την υποβολή των απαντήσεων, επικοινωνήστε τηλεφωνικά στο 22559656 με τον επικεφαλή συντονιστή της μελέτης: Δρ. Κωνσταντίνο Γιαννάκου (K.Giannakou@euc.ac.cy). Η παρούσα έρευνα δεν έχει λάβει καμία χρηματοδότηση και έχει εγκριθεί από την Εθνική Επιτροπή Βιοηθικής Κύπρου (ΕΕΒΚ ΕΠ 2020.01.82).

Συναινείτε να λάβετε μέρος στην μελέτη;
- Ναι
- Όχι

ΜΕΡΟΣ Α – ΔΗΜΟΓΡΑΦΙΚΑ ΣΤΟΙΧΕΙΑ

Α1. Ποιο είναι το φύλο σας;
- Άνδρας
- Γυναίκα
- Άλλο (Προσδιορίστε)

Α2. Ποια είναι η ηλικία σας;
- __________

Α3. Ποια είναι η χώρα καταγωγής σας;
- Κύπρος
- Ελλάδα
- Άλλο (Προσδιορίστε)

Α4. Σε ποια επαρχία κατοικείτε;
- Αθήνα
- Θεσσαλονίκη
- Λευκωσία
- Λάρνακα
- Λεμεσός
- Άλλο (Προσδιορίστε)

Α5. Σε τι περιοχή διαμένετε;
- Αστική
- Αγροτική

Α6. Ποιο είναι το επάγγελμά σας;
- Ιδιωτικός υπάλληλος
- Δημόσιος υπάλληλος
- Ελεύθερος/η επαγγελματίας
- Άνεργος/η
- Οικιακά
- Φοιτητής/τρια
- Συνταξιούχος/χα
- Άλλο (Προσδιορίστε)

Α7. Είστε επαγγελματίας υγείας (π.χ. γιατρός, νοσηλευτής, κλπ..);
- Ναι
- Όχι

Α8. Ποια η οικογενειακή σας κατάσταση;
- Έγγαμος-η
- Σε συμβίωση
- Άγαμος-η
- Διαζευγμένος-η
- Σε διάσταση
- Χήρος-α

Α9. Ποιο είναι το υψηλότερο επίπεδο εκπαίδευσης που έχετε ολοκληρώσει μέχρι τώρα;
- Δημοτικό

- Γυμνάσιο
- Λύκειο/Τεχνική Σχολή
- Κολλέγιο
- Πτυχίο/Προπτυχιακό
- Μάστερ/Μεταπτυχιακό
- Διδακτορικό

Α10. Ποιο είναι το θρήσκευμά σας;
- Χριστιανός Ορθόδοξος
- Χριστιανός Καθολικός
- Μουσουλμάνος
- Μάρτυρας του Ιεχωβά
- Άλλο (Προσδιορίστε)

Α11. Ποιο είναι το μηνιαίο σας ακαθάριστο εισόδημα (ευρώ);
- Δεν έχω εισόδημα
- Λιγότερο από 1001
- 1001-1500
- 1501-2000
- Περισσότερο από 2001

ΜΕΡΟΣ Β - ΣΤΟΙΧΕΙΑ ΓΙΑ ΤΗΝ ΚΑΤΑΣΤΑΣΗ ΕΜΒΟΛΙΑΣΜΟΥ

Β1. Είστε μονογονεϊκή οικογένεια;
- Ναι
- Όχι

Β2. Αριθμός ανήλικων παιδιών;
- _________

Β3. Προσδιορίστε το φύλο των παιδιών σας.
- 1^{ο} παιδί Αγόρι □ Κορίτσι □
- 2^{ο} παιδί Αγόρι □ Κορίτσι □
- 3^{ο} παιδί Αγόρι □ Κορίτσι □
- 4^{ο} παιδί Αγόρι □ Κορίτσι □
- 5^{ο} παιδί Αγόρι □ Κορίτσι □
- 6^{ο} παιδί Αγόρι □ Κορίτσι □

Β4. Ποιες είναι οι ηλικίες των παιδιών σας (για παράδειγμα, 1^{ο} παιδί =2 ετών, 2^{ο} παιδί =4 ετών κτλ.);
- _________

Β5. Ακολουθείτε πιστά τις καθορισμένες δόσεις όπως υποδεικνύονται από τις τοπικές συστάσεις για κάθε εμβόλιο;
- Ναι
- Όχι

ΜΕΡΟΣ Γ – ΓΕΝΙΚΕΣ ΓΝΩΣΕΙΣ ΓΙΑ ΤΟΝ ΕΜΒΟΛΙΑΣΜΟ

Γ1. Τα εμβόλια είναι περιττά, καθώς οι ιοί μπορούν να αντιμετωπιστούν με αντιβιοτικά.
- Σωστό
- Λάθος
- Δεν γνωρίζω

Γ2. Έχει αποδειχθεί η αποτελεσματικότητα των εμβολίων με επιδημιολογικές μελέτες.
- Σωστό
- Λάθος
- Δεν γνωρίζω

Γ3. Η συστηματική χορήγηση εμβολίων έχει συμβάλει στη μείωση ή και την εξάλειψη πολλών λοιμωδών νοσημάτων παγκοσμίως.
- Σωστό
- Λάθος
- Δεν γνωρίζω

Γ4. Μπορεί να γίνει εμβολιασμός το καλοκαίρι.
- Σωστό
- Λάθος
- Δεν γνωρίζω

Γ5. Μπορεί να γίνει εμβολιασμός όταν το παιδί μου έχει κρυολόγημα.
- Σωστό
- Λάθος
- Δεν γνωρίζω

Γ6. Μπορεί να γίνει εμβολιασμός όταν το παιδί μου έχει πυρετό (> 38° C).
- Σωστό
- Λάθος
- Δεν γνωρίζω

Γ7. Το εμβόλιο για την ιλαρά/ερυθρά/παρωτίτιδα (MMR) σχετίζεται με τον αυτισμό.
- Σωστό
- Λάθος
- Δεν γνωρίζω

Γ8. Τα παιδιά θα ήταν πιο ανθεκτικά εάν δεν εμβολιάζονταν.
- Σωστό
- Λάθος
- Δεν γνωρίζω

Γ9. Πολλά εμβόλια χορηγούνται πολύ νωρίς, με αποτέλεσμα το ανοσοποιητικό σύστημα των παιδιών να μην μπορεί να αναπτυχθεί.
- Σωστό
- Λάθος
- Δεν γνωρίζω

Γ10. Οι δόσεις των χημικών που χρησιμοποιούνται στα εμβόλια είναι επικίνδυνες για τον άνθρωπο.

- Σωστό
- Λάθος
- Δεν γνωρίζω

Γ11. Ο εμβολιασμός αυξάνει την εμφάνιση αλλεργιών.
- Σωστό
- Λάθος
- Δεν γνωρίζω

Γ12. Υπάρχει εμβόλιο για την πρόληψη του καρκίνου του τραχήλου της μήτρας.
- Σωστό
- Λάθος
- Δεν γνωρίζω

Γ13. Για νοσήματα που έχουν εξαφανιστεί δεν χρειάζεται να γίνεται εμβολιασμός.
- Σωστό
- Λάθος
- Δεν γνωρίζω

ΜΕΡΟΣ Δ - ΣΤΑΣΕΙΣ ΑΠΕΝΑΝΤΙ ΣΤΟΝ ΕΜΒΟΛΙΑΣΜΟ

Δ1. Πρέπει να πραγματοποιούνται όλοι οι εμβολιασμοί που προβλέπονται από το Εθνικό Πρόγραμμα Εμβολιασμών (Σχήμα Εμβολιασμών) στα παιδιά μας.
- Διαφωνώ απόλυτα
- Διαφωνώ
- Ούτε συμφωνώ ούτε διαφωνώ
- Συμφωνώ
- Συμφωνώ απόλυτα

Δ2. Όλα τα εμβόλια είναι ασφαλή.
- Διαφωνώ απόλυτα
- Διαφωνώ
- Ούτε συμφωνώ ούτε διαφωνώ
- Συμφωνώ
- Συμφωνώ απόλυτα

Δ3. Τα εμβόλια προστατεύουν τα παιδιά από σοβαρές και απειλητικές για τη ζωή ασθένειες.
- Διαφωνώ απόλυτα
- Διαφωνώ
- Ούτε συμφωνώ ούτε διαφωνώ
- Συμφωνώ
- Συμφωνώ απόλυτα

Δ4. Ο εμβολιασμός στην παιδική ηλικία προστατεύει δια βίου.
- Διαφωνώ απόλυτα
- Διαφωνώ
- Ούτε συμφωνώ ούτε διαφωνώ
- Συμφωνώ
- Συμφωνώ απόλυτα

Δ5. Ένα εμβόλιο παρέχει πάντα προστασία σε ένα παιδί.
- Διαφωνώ απόλυτα
- Διαφωνώ
- Ούτε συμφωνώ ούτε διαφωνώ
- Συμφωνώ
- Συμφωνώ απόλυτα

Δ6. Υπάρχουν πιθανές παρενέργειες από ορισμένα εμβόλια.
- Διαφωνώ απόλυτα
- Διαφωνώ
- Ούτε συμφωνώ ούτε διαφωνώ
- Συμφωνώ
- Συμφωνώ απόλυτα

Δ7. Τα εμβόλια μπορεί να προκαλέσουν προβλήματα σε βάθος χρόνου στα παιδιά.
- Διαφωνώ απόλυτα
- Διαφωνώ
- Ούτε συμφωνώ ούτε διαφωνώ
- Συμφωνώ
- Συμφωνώ απόλυτα

Δ8. Τα οφέλη από τον εμβολιασμό είναι περισσότερα από τους πιθανούς κινδύνους.
- Διαφωνώ απόλυτα
- Διαφωνώ
- Ούτε συμφωνώ ούτε διαφωνώ
- Συμφωνώ
- Συμφωνώ απόλυτα

Δ9. Ο μεγάλος αριθμός εμβολίων μπορεί να επηρεάσει αρνητικά το ανοσοποιητικό σύστημα ενός παιδιού.
- Διαφωνώ απόλυτα
- Διαφωνώ
- Ούτε συμφωνώ ούτε διαφωνώ
- Συμφωνώ
- Συμφωνώ απόλυτα

Δ10. Τα παιδιά πρέπει να εμβολιάζονται αμέσως μετά την κυκλοφορία ενός νέου εμβολίου.
- Διαφωνώ απόλυτα
- Διαφωνώ
- Ούτε συμφωνώ ούτε διαφωνώ
- Συμφωνώ
- Συμφωνώ απόλυτα

Δ11. Αμφιβάλλω για την ασφάλεια και την αποτελεσματικότητα των νέων εμβολίων.
- Διαφωνώ απόλυτα
- Διαφωνώ
- Ούτε συμφωνώ ούτε διαφωνώ
- Συμφωνώ
- Συμφωνώ απόλυτα

Δ12. Πιστεύω στην χρησιμότητα των εμβολίων.
- Διαφωνώ απόλυτα
- Διαφωνώ
- Ούτε συμφωνώ ούτε διαφωνώ
- Συμφωνώ
- Συμφωνώ απόλυτα

Δ13. Ορισμένα εμβόλια γίνονται με γνώμονα την εμπορική σκοπιμότητα.
- Διαφωνώ απόλυτα
- Διαφωνώ
- Ούτε συμφωνώ ούτε διαφωνώ
- Συμφωνώ
- Συμφωνώ απόλυτα

Δ14. Θεωρώ πως η φυσική παιδική ασθένεια είναι καλύτερη από τον εμβολιασμό.
- Διαφωνώ απόλυτα
- Διαφωνώ
- Ούτε συμφωνώ ούτε διαφωνώ
- Συμφωνώ
- Συμφωνώ απόλυτα

ΜΕΡΟΣ Ε - ΠΗΓΕΣ ΠΛΗΡΟΦΟΡΙΩΝ ΚΑΙ ΓΕΝΙΚΕΣ ΕΡΩΤΗΣΕΙΣ ΣΧΕΤΙΚΑ ΜΕ ΤΟΝ ΕΜΒΟΛΙΑΣΜΟ

Ε1. Είμαι επαρκώς ενημερωμένος/η σε θέματα εμβολιασμού
- Διαφωνώ απόλυτα
- Διαφωνώ
- Ούτε συμφωνώ ούτε διαφωνώ
- Συμφωνώ
- Συμφωνώ απόλυτα

Ε2. Ποια είναι η κύρια πηγή ενημέρωσης για εσάς για θέματα που αφορούν τον εμβολιασμό των παιδιών σας;
- Παιδίατρος
- Φαρμακοποιός
- Οικογενειακός Ιατρός
- Προσωπικός Ιατρός
- Διαδίκτυο και Μέσα Μαζικής Ενημέρωσης (ΜΜΕ)
- Οικογένεια και φίλοι
- Άλλο (Προσδιορίστε)

Ε3. Εμπιστεύομαι απόλυτα τον παιδίατρο του παιδιού μου.
- Διαφωνώ απόλυτα
- Διαφωνώ
- Ούτε συμφωνώ ούτε διαφωνώ
- Συμφωνώ
- Συμφωνώ απόλυτα

Ε4. Συζητώ ελεύθερα τις ανησυχίες μου με τον παιδίατρο.
- Διαφωνώ απόλυτα
- Διαφωνώ
- Ούτε συμφωνώ ούτε διαφωνώ
- Συμφωνώ
- Συμφωνώ απόλυτα

Ε5. Έχετε καθυστερήσει ποτέ τον εμβολιασμό του παιδιού/παιδιών σας;
- Ναι
- Όχι

Ε6. Αν έχετε καθυστερήσει τον εμβολιασμό του παιδιού/παιδιών σας, ποιοι ήταν οι κύριοι λόγοι;
- Ασθένεια
- Έλλειψη ξεκάθαρων πληροφοριών
- Πρόταση του παιδιάτρου
- Φόβος για παρενέργειες του εμβολίου
- Αυξημένο κόστος εμβολίων
- Αυξημένο κόστος ιατρικής επίσκεψης
- Μεγάλη απόσταση από τον τόπο εμβολιασμού
- Άλλο (Προσδιορίστε)

Ε7. Εμβολιαστήκατε με κάποιο εμβόλιο κατά την διάρκεια της εγκυμοσύνη σας (αφορά μόνο γυναίκες);
- Ναι
- Όχι
