# Supplementary figures and images for: Attitudes and perceptions of mothers towards childhood vaccination in Greece: lessons to improve the childhood COVID-19 vaccination acceptance
Source: Front Pediatr. 2022 Aug 25;10:951039. doi: 10.3389/fped.2022.951039 (PMC9453258; doi:10.3389/fped.2022.951039)

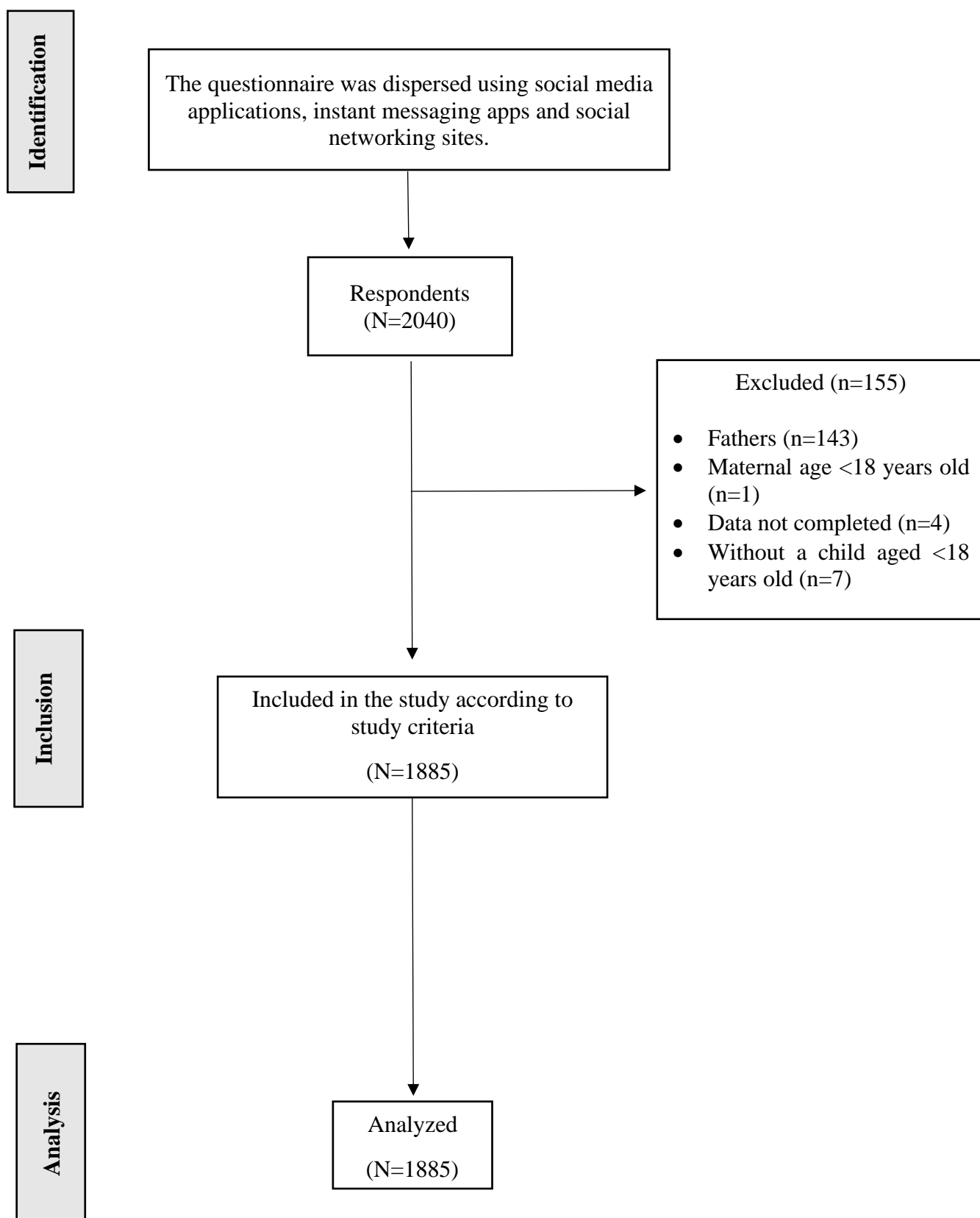

**Supplementary Figure 1.** Study flow diagram

Supplement: Supplementary file 2 [file Image_1.pdf]
