# Supplementary material for: Attitudes and perceptions of mothers towards childhood vaccination in Greece: lessons to improve the childhood COVID-19 vaccination acceptance
Source: Front Pediatr. 2022 Aug 25;10:951039. doi: 10.3389/fped.2022.951039 (PMC9453258; doi:10.3389/fped.2022.951039)
Supplement: Supplementary file 3 [file Table_1.pdf]

Supplementary Table 1. Maternal responses to questions about vaccination attitudes by age of mother and their child/children.

	Age of mother (SD)	p-value[a]	Age of children (IQR)	p-value[b]
**Q1. All vaccinations provided by the National Vaccination Program must be offered to our children.**				
**D**	36.5 (5.6)		48 (24-75)	
**N**	36.4 (5.4)	0.89	48 (30-72)	0.68
**A**	36.3 (5.0)		48 (24-72)	
**Q2. All vaccines are safe.**				
**D**	36.5 (5.4)		48 (24-72)	
**N**	35.7 (5.3)	<0.01	48 (24-72)	0.55
**A**	36.6 (4.7)		48 (24-72)	
**Q3. Vaccines protect children from serious and life-threatening diseases.**				
**D**	35.6 (5.7)		39 (24-72)	
**N**	35.6 (6.2)	0.20	48 (30-84)	0.49
**A**	36.3 (4.9)		48 (24-72)	
**Q4. Vaccination in childhood protects for a lifetime.**				
**D**	35.8 (5.0)		48 (24-72)	
**N**	36.2 (4.8)	0.03	48 (24-72)	0.86
**A**	36.6 (5.2)		48 (24-72)	
**Q5. A vaccine always provides protection to a child.**				
**D**	35.5 (5.0)		48 (24-72)	
**N**	35.9 (5.0)	**<0.01**	48 (24-72)	0.81
**A**	36.9 (5.0)		48 (24-72)	
**Q6. There are possible side effects from some vaccines.**				
**D**	38.5 (4.4)		42 (24-72)	
**N**	36.6 (5.7)	**0.01**	48 (24-72)	0.44
**A**	36.2 (5.0)		48 (24-72)	
**Q7. Vaccines can cause long-term problems in children.**				
**D**	36.3 (5.0)		48 (24-72)	
**N**	36.2 (5.0)	0.97	48 (24-72)	0.87
**A**	36.3 (5.5)		48 (24-72)	
**Q8. The benefits of vaccination outweigh the potential risks.**				
**D**	36.6 (5.8)		48 (30-72)	
**N**	36.2 (5.9)	0.89	39 (24-72)	0.67
**A**	36.3 (4.9)		48 (24-72)	
**Q9. Large number of vaccines can adversely affect the immune system of children.**				
**D**	36.3 (5.0)		48 (24-72)	
**N**	36.2 (4.9)	0.89	48 (24-72)	0.64
**A**	36.4 (5.9)		36 (24-72)	
**Q10. Children should be vaccinated immediately after the release of a new vaccine.**				
**D**	35.9 (5.1)	<0.01	48 (24-72)	0.64
**N**	36.7 (4.8)		48 (24-72)	

**A**	37.4 (4.7)		48 (24-72)	
**Q11. I doubt the safety and effectiveness of new vaccines.**				
**D**	36.8 (4.7)		48 (24-72)	
**N**	36.2 (4.8)	**0.02**	48 (24-72)	**0.04**
**A**	35.9 (5.5)		37.5 (24-69)	
**Q12. I believe in the usefulness of vaccines.**				
**D**	36.0 (5.6)		42 (24-72)	
**N**	36.4 (6.0)	0.94	48 (24-72)	0.85
**A**	36.3 (5.0)		48 (24-72)	
**Q13. Some vaccines are made for commercial purposes.**				
**D**	36.8 (4.6)		48 (24-72)	
**N**	36.2 (5.0)	**0.02**	48 (24-72)	0.06
**A**	36.0 (5.3)		42 (24-72)	
**Q14. I think natural childhood illness is better than vaccination.**				
**D**	36.3 (4.9)		48 (24-72)	
**N**	36.0 (5.4)	0.06	42 (24-72)	0.40
**A**	37.2 (5.6)		48 (24-72)	

Abbreviations: D, absolutely disagree/disagree; N, neither disagree nor agree; A, agree/absolutely agree; SD, standard deviation; IQR, interquartile range; [a] One-way analysis of variance (ANOVA); [b] Kruskal–Wallis rank test; Bold font indicates statistical significance after a Bonferroni correction ($p<0.05$).
