# Supplementary material for: Attitudes and perceptions of mothers towards childhood vaccination in Greece: lessons to improve the childhood COVID-19 vaccination acceptance
Source: Front Pediatr. 2022 Aug 25;10:951039. doi: 10.3389/fped.2022.951039 (PMC9453258; doi:10.3389/fped.2022.951039)
Supplement: Supplementary file 4 [file Table_2.pdf]

Supplementary Table 2. Maternal responses to questions about vaccination attitudes by geographical region and area of residence

	Geographical region of residence					p-value[a]	Area of residence			p-value[a]
	Total	Attica	Central Greece	North Greece	Crete/Aegean Islands		Total	Urban	Rural	
**Q1. All vaccinations provided by the National Vaccination Program must be offered to our children.**										
**D**	80 (4.3)	39 (4.4)	12 (4.9)	20 (3.7)	9 (4.3)		79 (4.4)	69 (4.4)	10 (4.3)	
**N**	102 (5.4)	45 (5.1)	14 (5.7)	35 (6.4)	8 (3.8)	0.80	97 (5.3)	84 (5.3)	13 (5.5)	1.00
**A**	1703 (90.3)	799 (90.5)	219 (89.4)	491 (89.9)	194 (91.9)		1636 (90.3)	1424 (90.3)	212 (90.2)	
**Q2. All vaccines are safe.**										
**D**	224 (11.9)	104 (11.8)	35 (14.3)	66 (12.1)	19 (9.0)		218 (12.0)	189 (12.0)	29 (12.4)	
**N**	669 (35.5)	285 (32.3)	93 (37.9)	208 (38.2)	83 (39.3)	0.07	645 (35.6)	547 (34.7)	98 (41.9)	0.07
**A**	991 (52.6)	494 (55.9)	117 (47.8)	271 (49.7)	109 (51.7)		948 (52.4)	841 (53.3)	107 (45.7)	
**Q3. Vaccines protect children from serious and life-threatening diseases.**										
**D**	40 (2.1)	15 (1.7)	8 (3.3)	9 (1.6)	8 (3.8)		39 (2.1)	33 (2.1)	6 (2.6)	
**N**	116 (6.2)	44 (5.0)	15 (6.1)	44 (8.1)	13 (6.2)	0.08	112 (6.2)	97 (6.1)	15 (6.4)	0.89
**A**	1727 (91.7)	822 (93.3)	222 (90.6)	493 (90.3)	190 (90.0)		1659 (91.7)	1446 (91.8)	213 (91.0)	
**Q4. Vaccination in childhood protects for a lifetime.**										
**D**	406 (21.5)	200 (22.7)	50 (20.4)	116 (21.2)	40 (18.9)		394 (21.8)	354 (22.5)	40 (17.0)	
**N**	572 (30.4)	258 (29.3)	72 (29.4)	160 (29.3)	82 (38.9)	0.17	542 (29.9)	471 (29.9)	71 (30.2)	0.14
**A**	905 (48.1)	423 (48.0)	123 (50.2)	270 (49.5)	89 (42.2)		874 (48.3)	750 (47.6)	124 (52.8)	
**Q5. A vaccine always provides protection to a child.**										
**D**	390 (20.7)	173 (19.6)	55 (22.4)	118 (21.6)	44 (20.9)		377 (20.8)	335 (21.3)	42 (17.9)	
**N**	554 (29.4)	248 (28.1)	71 (29.0)	158 (29.0)	77 (36.7)	0.17	530 (29.3)	456 (28.9)	74 (31.5)	0.45
**A**	939 (49.9)	462 (52.3)	119 (48.6)	269 (49.4)	89 (42.4)		903 (49.9)	784 (49.8)	119 (50.6)	
**Q6. There are possible side effects from some vaccines.**										
**D**	37 (2.0)	13 (1.5)	8 (3.3)	10 (1.8)	6 (2.8)		32 (1.8)	28 (1.8)	4 (1.7)	
**N**	208 (11.0)	109 (12.3)	24 (9.8)	55 (10.1)	20 (9.5)	0.33	202 (11.1)	175 (11.1)	27 (11.5)	0.98
**A**	1640 (87.0)	761 (86.2)	213 (86.9)	481 (88.1)	185 (87.7)		1578 (87.1)	1374 (87.1)	204 (86.8)	
**Q7. Vaccines can cause long-term problems in children.**										
**D**	929 (49.3)	474 (53.7)	112 (45.7)	247 (45.2)	96 (45.5)		888 (49.0)	787 (49.9)	101 (43.0)	
**N**	765 (40.6)	328 (37.1)	106 (43.3)	238 (43.6)	93 (44.1)	**0.04**	739 (40.8)	632 (40.1)	107 (45.5)	0.14
**A**	191 (10.1)	81 (9.2)	27 (11.0)	61 (11.2)	22 (10.4)		185 (10.2)	158 (10.0)	27 (11.5)	
**Q8. The benefits of vaccination outweigh the potential risks.**										
**D**	49 (2.6)	25 (2.8)	6 (2.4)	10 (1.8)	8 (3.8)		48 (2.6)	44 (2.8)	4 (1.7)	
**N**	155 (8.2)	71 (8.1)	18 (7.4)	49 (9.0)	17 (8.1)	0.77	150 (8.3)	128 (8.1)	22 (9.4)	0.52
**A**	1680 (89.2)	787 (89.1)	221 (90.2)	487 (89.2)	185 (88.1)		1613 (89.1)	1404 (89.1)	209 (88.9)	
**Q9. Large number of vaccines can adversely affect the immune system of children.**										
**D**	1160 (61.7)	564 (64.0)	155 (63.5)	325 (59.5)	116 (55.0)		1114 (61.6)	976 (62.0)	138 (58.7)	
**N**	554 (29.4)	244 (27.7)	66 (27.1)	166 (30.4)	78 (36.9)	0.13	531 (29.3)	455 (28.9)	76 (32.4)	0.55
**A**	168 (8.9)	73 (8.3)	23 (9.4)	55 (10.1)	17 (8.1)		164 (9.1)	143 (9.1)	21 (8.9)	
**Q10. Children should be vaccinated immediately after the release of a new vaccine.**										
**D**	1151 (61.2)	512 (58.1)	165 (67.3)	335 (61.6)	139 (65.9)	0.12	1108 (61.3)	950 (60.4)	158 (67.2)	**0.03**

**N**	637 (33.9)	321 (36.4)	71 (29.0)	184 (33.8)	61 (28.9)		612 (33.8)	550 (35.0)	62 (26.4)	
**A**	93 (4.9)	48 (5.5)	9 (3.7)	25 (4.6)	11 (5.2)		88 (4.9)	73 (4.6)	15 (6.4)	
**Q11. I doubt the safety and effectiveness of new vaccines.**										
**D**	441 (23.4)	220 (24.9)	51 (20.8)	124 (22.7)	46 (21.8)		421 (23.3)	373 (23.7)	48 (20.4)	
**N**	895 (47.5)	422 (47.9)	105 (42.9)	260 (47.6)	108 (51.2)	0.15	864 (47.7)	748 (47.4)	116 (49.4)	0.55
**A**	548 (29.1)	240 (27.2)	89 (36.3)	162 (29.7)	57 (27.0)		526 (29.0)	455 (28.9)	71 (30.2)	
**Q12. I believe in the usefulness of vaccines.**										
**D**	41 (2.2)	14 (1.6)	10 (4.1)	9 (1.7)	8 (3.8)		40 (2.2)	35 (2.2)	5 (2.1)	
**N**	114 (6.0)	48 (5.4)	11 (4.5)	40 (7.3)	15 (7.1)	0.05	109 (6.0)	92 (5.8)	17 (7.2)	0.70
**A**	1728 (91.8)	820 (93.0)	224 (91.4)	496 (91.0)	188 (89.1)		1661 (91.8)	1448 (92.0)	213 (90.7)	
**Q13. Some vaccines are made for commercial purposes.**										
**D**	465 (24.7)	248 (28.1)	57 (23.2)	120 (22.0)	40 (19.0)		449 (24.8)	398 (25.3)	51 (21.7)	
**N**	747 (39.7)	337 (38.3)	94 (38.4)	216 (39.6)	100 (47.4)	**0.02**	716 (39.6)	620 (39.4)	96 (40.8)	0.49
**A**	671 (35.6)	296 (33.6)	94 (38.4)	210 (38.4)	71 (33.6)		645 (35.6)	557 (35.3)	88 (37.5)	
**Q14. I think natural childhood illness is better than vaccination.**										
**D**	1355 (72.0)	658 (74.5)	175 (72.0)	381 (69.9)	141 (66.8)		1306 (72.2)	1152 (73.2)	154 (65.5)	
**N**	404 (21.5)	171 (19.4)	50 (20.6)	129 (23.7)	54 (25.6)	0.27	382 (21.1)	321 (20.4)	61 (26.0)	0.05
**A**	123 (6.5)	54 (6.1)	18 (7.4)	35 (6.4)	16 (7.6)		121 (6.7)	101 (6.4)	20 (8.5)	

Abbreviations: D, absolutely disagree/disagree; N, neither disagree nor agree; A, agree/absolutely agree; [a]Differences were tested using chi2 test; Bold font indicates statistical significance after a Bonferroni correction ($p<0.05$).
