# Supplementary material for: Attitudes and perceptions of mothers towards childhood vaccination in Greece: lessons to improve the childhood COVID-19 vaccination acceptance
Source: Front Pediatr. 2022 Aug 25;10:951039. doi: 10.3389/fped.2022.951039 (PMC9453258; doi:10.3389/fped.2022.951039)
Supplement: Supplementary file 5 [file Table_3.pdf]

**Supplementary Table 3.** Maternal responses to questions about vaccination attitudes by questions related to previous vaccination behaviors.

| | Do you strictly adhere to the prescribed dosage as indicated by the local recommendations for each vaccine? | | | | Have you ever delayed your child/children vaccination? | | | | Have you vaccinated during your pregnancy? | | | |
|---|---|---|---|---|---|---|---|---|---|---|---|---|
| | **Total** | **No** | **Yes** | **p-value**[a] | **Total** | **No** | **Yes** | **p-value**[a] | **Total** | **No** | **Yes** | **p-value**[a] |
| **Q1. All vaccinations provided by the National Vaccination Program must be offered to our children.** | | | | | | | | | | | | |
| **D** | 76 (4.0) | 57 (53.3) | 19 (1.1) | **<0.01** | 75 (4.0) | 14 (1.5) | 61 (6.3) | **<0.01** | 80 (4.3) | 78 (5.5) | 2 (0.4) | **<0.01** |
| **N** | 101 (5.4) | 28 (26.2) | 73 (4.1) | | 101 (5.4) | 27 (3.0) | 74 (7.7) | | 102 (5.4) | 92 (6.5) | 10 (2.2) | |
| **A** | 1700 (90.6) | 22 (20.5) | 1678 (94.8) | | 1700 (90.6) | 869 (95.5) | 831 (86.0) | | 1697 (90.3) | 1253 (88.0) | 444 (97.4) | |
| **Q2. All vaccines are safe.** | | | | | | | | | | | | |
| **D** | 218 (11.6) | 87 (81.3) | 131 (7.4) | **<0.01** | 218 (11.6) | 59 (6.5) | 159 (16.5) | **<0.01** | 223 (11.9) | 203 (14.3) | 20 (4.4) | **<0.01** |
| **N** | 668 (35.6) | 17 (15.9) | 651 (36.8) | | 668 (35.6) | 317 (34.9) | 351 (36.3) | | 668 (35.6) | 538 (37.8) | 130 (28.6) | |
| **A** | 990 (52.8) | 3 (2.8) | 987 (55.8) | | 989 (52.8) | 533 (58.6) | 456 (47.2) | | 987 (52.5) | 682 (47.9) | 305 (67.0) | |
| **Q3. Vaccines protect children from serious and life-threatening diseases.** | | | | | | | | | | | | |
| **D** | 36 (1.9) | 27 (25.2) | 9 (0.5) | **<0.01** | 36 (1.9) | 7 (0.8) | 29 (3.0) | **<0.01** | 40 (2.1) | 39 (2.7) | 1 (0.2) | **<0.01** |
| **N** | 114 (6.1) | 35 (32.7) | 79 (4.5) | | 115 (6.1) | 30 (3.3) | 85 (8.8) | | 116 (6.2) | 108 (7.6) | 8 (1.8) | |
| **A** | 1725 (92.0) | 45 (42.1) | 1680 (95.0) | | 1723 (92.0) | 871 (95.9) | 852 (88.2) | | 1721 (91.7) | 1274 (89.7) | 447 (98.0) | |
| **Q4. Vaccination in childhood protects for a lifetime.** | | | | | | | | | | | | |
| **D** | 401 (21.4) | 69 (64.5) | 332 (18.8) | **<0.01** | 400 (21.3) | 148 (16.3) | 252 (26.1) | **<0.01** | 404 (21.5) | 319 (22.5) | 85 (18.6) | 0.16 |
| **N** | 570 (30.4) | 33 (30.8) | 537 (30.4) | | 571 (30.5) | 270 (29.7) | 301 (31.2) | | 571 (30.4) | 434 (30.5) | 137 (30.1) | |
| **A** | 904 (48.2) | 5 (4.7) | 899 (50.8) | | 903 (48.2) | 491 (54.0) | 412 (42.7) | | 902 (48.1) | 668 (47.0) | 234 (51.3) | |
| **Q5. A vaccine always provides protection to a child.** | | | | | | | | | | | | |
| **D** | 384 (20.5) | 80 (74.8) | 304 (17.2) | **<0.01** | 384 (20.5) | 144 (15.8) | 240 (24.9) | **<0.01** | 389 (20.7) | 311 (21.9) | 78 (17.1) | **0.02** |
| **N** | 552 (29.4) | 23 (21.5) | 529 (29.9) | | 553 (29.5) | 254 (27.9) | 299 (31.0) | | 552 (29.4) | 427 (30.0) | 125 (27.4) | |
| **A** | 939 (52.1) | 4 (3.7) | 935 (52.9) | | 937 (50.0) | 511 (56.3) | 426 (44.1) | | 936 (49.9) | 683 (48.1) | 253 (55.5) | |
| **Q6. There are possible side effects from some vaccines.** | | | | | | | | | | | | |
| **D** | 37 (2.0) | 0 (0.0) | 37 (2.1) | **<0.01** | 37 (2.0) | 24 (2.6) | 13 (1.4) | **0.03** | 37 (2.0) | 31 (2.2) | 6 (1.3) | 0.15 |
| **N** | 208 (11.1) | 2 (1.9) | 206 (11.6) | | 208 (11.1) | 112 (12.3) | 96 (9.9) | | 208 (11.1) | 148 (10.4) | 60 (13.2) | |
| **A** | 1632 (86.9) | 105 (98.1) | 1527 (86.3) | | 1631 (86.9) | 774 (85.1) | 857 (88.7) | | 1634 (86.9) | 1244 (87.4) | 390 (85.5) | |
| **Q7. Vaccines can cause long-term problems in children.** | | | | | | | | | | | | |
| **D** | 929 (49.5) | 3 (2.8) | 926 (52.3) | **<0.01** | 928 (49.5) | 502 (55.2) | 426 (44.1) | **<0.01** | 924 (49.2) | 656 (46.1) | 268 (58.8) | **<0.01** |
| **N** | 762 (40.6) | 21 (19.6) | 741 (41.9) | | 763 (40.7) | 351 (38.6) | 412 (42.6) | | 764 (40.7) | 596 (41.9) | 168 (36.8) | |
| **A** | 186 (9.9) | 83 (77.6) | 103 (5.8) | | 185 (9.8) | 57 (6.2) | 128 (13.3) | | 191 (10.1) | 171 (12.0) | 20 (4.4) | |
| **Q8. The benefits of vaccination outweigh the potential risks.** | | | | | | | | | | | | |
| **D** | 46 (2.4) | 34 (31.8) | 12 (0.7) | **<0.01** | 45 (2.4) | 7 (0.8) | 38 (3.9) | **<0.01** | 49 (2.6) | 46 (3.2) | 3 (0.7) | **<0.01** |
| **N** | 153 (8.2) | 43 (40.2) | 110 (6.2) | | 153 (8.2) | 52 (5.7) | 101 (10.5) | | 155 (8.3) | 138 (9.7) | 17 (3.7) | |
| **A** | 1677 (89.4) | 30 (28.0) | 1647 (93.1) | | 1677 (89.4) | 850 (93.5) | 827 (85.6) | | 1674 (89.1) | 1238 (87.1) | 436 (95.6) | |
| **Q9. Large number of vaccines can adversely affect the immune system of children.** | | | | | | | | | | | | |
| **D** | 1159 (61.8) | 8 (7.5) | 1151 (65.1) | **<0.01** | 1158 (61.8) | 612 (67.3) | 546 (56.6) | **<0.01** | 1155 (61.6) | 826 (58.1) | 329 (72.5) | **<0.01** |

| | | | | | | | | | | | | |
|---|---|---|---|---|---|---|---|---|---|---|---|---|
| **N** | 552 (29.5) | 20 (18.7) | 532 (30.1) | | 554 (29.6) | 257 (28.3) | 297 (30.8) | | 554 (29.5) | 444 (31.2) | 110 (24.2) | |
| **A** | 163 (8.7) | 79 (73.8) | 84 (4.8) | | 161 (8.6) | 40 (4.4) | 121 (12.6) | | 167 (8.9) | 152 (10.7) | 15 (3.3) | |
| **Q10. Children should be vaccinated immediately after the release of a new vaccine.** | | | | | | | | | | | | |
| **D** | 1144 (61.0) | 105 (98.1) | 1039 (58.8) | **<0.01** | 1144 (61.1) | 502 (55.4) | 642 (66.5) | **<0.01** | 1147 (61.2) | 920 (64.8) | 227 (49.8) | **<0.01** |
| **N** | 636 (34.0) | 2 (1.9) | 634 (35.9) | | 635 (33.9) | 343 (37.8) | 292 (30.3) | | 635 (33.9) | 446 (31.4) | 189 (41.4) | |
| **A** | 93 (5.0) | 0 (0.0) | 93 (5.3) | | 93 (5.0) | 62 (6.8) | 31 (3.2) | | 93 (4.9) | 53 (3.8) | 40 (8.8) | |
| **Q11. I doubt the safety and effectiveness of new vaccines.** | | | | | | | | | | | | |
| **D** | 441 (23.5) | 2 (1.9) | 439 (24.8) | **<0.01** | 441 (23.5) | 253 (27.8) | 188 (19.5) | **<0.01** | 440 (23.4) | 298 (21.0) | 142 (31.1) | **<0.01** |
| **N** | 893 (47.6) | 16 (15.0) | 877 (49.6) | | 892 (47.6) | 446 (49.1) | 446 (46.2) | | 891 (47.4) | 663 (46.6) | 228 (50.0) | |
| **A** | 542 (28.9) | 89 (83.1) | 453 (25.6) | | 542 (28.9) | 210 (23.1) | 332 (34.3) | | 547 (29.2) | 461 (32.4) | 86 (18.9) | |
| **Q12. I believe in the usefulness of vaccines.** | | | | | | | | | | | | |
| **D** | 37 (2.0) | 27 (25.2) | 10 (0.6) | **<0.01** | 37 (2.0) | 6 (0.7) | 31 (3.2) | **<0.01** | 41 (2.2) | 41 (2.9) | 0 (0.0) | **<0.01** |
| **N** | 112 (6.0) | 38 (35.5) | 74 (4.2) | | 113 (6.0) | 34 (3.7) | 79 (8.2) | | 113 (6.0) | 105 (7.4) | 8 (1.7) | |
| **A** | 1727 (92.0) | 42 (39.3) | 1685 (95.2) | | 1724 (92.0) | 869 (95.6) | 855 (88.6) | | 1723 (91.8) | 1275 (89.7) | 448 (98.3) | |
| **Q13. Some vaccines are made for commercial purposes.** | | | | | | | | | | | | |
| **D** | 465 (24.8) | 1 (0.9) | 464 (26.2) | **<0.01** | 463 (24.7) | 260 (28.6) | 203 (21.0) | **<0.01** | 464 (24.7) | 315 (22.1) | 149 (32.7) | **<0.01** |
| **N** | 746 (39.8) | 9 (8.4) | 737 (41.7) | | 746 (39.8) | 370 (40.7) | 376 (39.0) | | 746 (39.7) | 550 (38.7) | 196 (43.0) | |
| **A** | 664 (35.4) | 97 (90.7) | 567 (32.1) | | 665 (35.5) | 279 (30.7) | 386 (40.0) | | 668 (35.6) | 557 (39.2) | 111 (24.3) | |
| **Q14. I think natural childhood illness is better than vaccination.** | | | | | | | | | | | | |
| **D** | 1353 (72.2) | 12 (11.3) | 1341 (75.8) | **<0.01** | 1352 (72.2) | 685 (75.4) | 667 (69.2) | **<0.01** | 1351 (72.0) | 980 (69.0) | 371 (81.4) | **<0.01** |
| **N** | 404 (21.6) | 33 (31.1) | 371 (21.0) | | 403 (21.5) | 191 (21.0) | 212 (22.0) | | 402 (21.4) | 327 (23.0) | 75 (16.4) | |
| **A** | 117 (6.2) | 61 (57.6) | 56 (3.2) | | 118 (6.3) | 33 (3.6) | 85 (8.8) | | 123 (6.6) | 113 (8.0) | 10 (2.2) | |

Abbreviations: D, absolutely disagree/disagree; N, neither disagree nor agree; A, agree/absolutely agree; [a]Differences were tested using chi2 test; Bold font indicates statistical significance after a Bonferroni correction (p<0.05).
